# Pollicott-Ruelle Resonant States and Betti Numbers

**DOI:** 10.1007/s00220-020-03793-2

**Published:** 2020-07-22

**Authors:** Benjamin Küster, Tobias Weich

**Affiliations:** 1 Université Paris-Saclay, CNRS, Orsay France; 2 Universität Paderborn, Paderborn, Germany

## Abstract

Given a closed orientable hyperbolic manifold of dimension $\ne 3$ we prove that the multiplicity of the Pollicott-Ruelle resonance of the geodesic flow on perpendicular one-forms at zero agrees with the first Betti number of the manifold. Additionally, we prove that this equality is stable under small perturbations of the Riemannian metric and simultaneous small perturbations of the geodesic vector field within the class of contact vector fields. For more general perturbations we get bounds on the multiplicity of the resonance zero on all one-forms in terms of the first and zeroth Betti numbers. Furthermore, we identify for hyperbolic manifolds further resonance spaces whose multiplicities are given by higher Betti numbers.

## Introduction

Pollicott-Ruelle resonances have been introduced in the 1980’s in order to study mixing properties of hyperbolic flows and can nowadays be understood as a discrete spectrum of the generating vector field (see Sect. 1.2 for a definition and references). Very recently it has been discovered that in certain cases some particular Pollicott-Ruelle resonances have a topological meaning. Let us recall these results:

In [DZ17] Dyatlov and Zworski prove that on a closed orientable surface ${\mathcal {M}}$ of negative curvature the Ruelle zeta function at zero vanishes to the order $|\chi (\mathcal M)|$, where $\chi ({\mathcal {M}})$ is the Euler characteristic of ${{\mathcal {M}}}$, generalizing a result of Fried in constant curvature [Fri86].^1^ Dyatlov and Zworski prove their result as follows: By previous results on the meromorphic continuation of the Ruelle zeta function (see [DZ16, GLP13]) the order of vanishing of the Ruelle zeta function at zero can be expressed as the alternating sum $\sum _{k=0}^2 (-1)^ {k+1} m_{{\mathcal {L}}_X, \Lambda ^k X^\perp }(0)$, where $m_{{\mathcal {L}}_X, \Lambda ^k X^\perp }(0)$ is the multiplicity of the resonance zero of the Lie derivative ${\mathcal {L}}_X$ along the geodesic vector field $X\in \Gamma ^\infty (T(S^*{{\mathcal {M}}}))$ acting on perpendicular *k*-forms. The latter are those *k*-forms on the unit co-sphere bundle $S^*{{\mathcal {M}}}$ that vanish upon contraction with *X* (for the precise definition of the multiplicities, see Sects. 1.1 and 1.2). For closed orientable surfaces it is rather easy to see that $m_{{\mathcal {L}}_X, \Lambda ^0 X^\perp }(0) = m_{{\mathcal {L}}_X, \Lambda ^2 X^\perp }(0)= b_0({\mathcal {M}})=b_2({\mathcal {M}})$, thus the central task is to prove that $m_{{\mathcal {L}}_X, X^\perp }(0)= b_1({\mathcal {M}})$. Dyatlov and Zworski achieve this by combining microlocal analysis with Hodge theory [DZ17, Proposition 3.1(2)]. This is a remarkable result also apart from its implications on zeta function questions because it identifies a resonance whose multiplicity has a precise topological meaning.

Let us mention a second result establishing a connection between Pollicott-Ruelle resonances and topology: Dang and Rivière [DR19c] examine a general Anosov flow $\varphi _t= e^{Yt}$ on a closed orientable manifold. The Lie derivative ${\mathcal {L}}_Y$ has a discrete spectrum (the Pollicott-Ruelle spectrum) on certain spaces of anisotropic *p*-currents and it is shown that the exterior derivative acting on generalized eigenspaces of the eigenvalue zero forms a complex which is quasi-isomorphic to the de Rham complex.^2^ While this result gives no precise information about the multiplicities of the resonances, it gives lower bounds for them and it holds in very great generality.

As a third result we would like to mention [GHW18] where the relation between Pollicott-Ruelle and quantum resonances is studied for compact and convex co-compact hyperbolic surfaces. For this correspondence the resonances at negative integers turn out to be exceptional points and it is shown that their multiplicities can be expressed by the Euler characteristic of the hyperbolic surface. The proof uses a Poisson transform to establish a bijection between the resonant states and holomorphic sections of certain line bundles, and the formula for the multiplicities follows from a Riemann-Roch theorem.

In the present article we broaden the picture regarding the topological properties of Pollicott-Ruelle resonant states. To this end, we combine some of the above approaches: In a first step we use a quantum-classical correspondence to find new examples of resonances with topological multiplicities. In particular, we prove

### Proposition 0.1

For any closed orientable hyperbolic manifold ${\mathcal {M}}$ of dimension $n+1$ with $n\ne 2$, one has

$$\begin{aligned} m_{{\mathcal {L}}_X, X^\perp }(0)= b_1({\mathcal {M}}). \end{aligned}$$

Furthermore, the resonance zero has no Jordan block and if $n\ge 3$, then zero is the unique leading resonance and there is a spectral gap.^3^

We prove these statements using the general framework of vector-valued quantum-classical correspondence developed by the authors [KW19] as well as a Poisson transform of Gaillard [Gai86].^4^ Without any further effort these ingredients provide additional examples of resonance multiplicities related to not only the first but to all Betti numbers, see Proposition 2.3. More precisely, the latter result shows that the *p*-th Betti number of a closed orientable hyperbolic manifold can be recovered as the dimension of the space of some particular resonant *p*-forms in the kernel of a so-called *horocycle operator* (see Sect. 2.2). For $n=1$ the first statement in Proposition 0.1 is the special case of [DZ17, Proposition 3.1(2)] restricted to hyperbolic surfaces. Interestingly $n=2$ is an exceptional case and the multiplicity is given by $m_{{\mathcal {L}}_X, X^\perp }(0)= 2b_1({\mathcal {M}})$ (see Remark 2.2). For $n> 2$ the statement can be considered as a generalization of the Dyatlov-Zworski result to higher dimensions at the cost of restricting to manifolds of constant negative curvature.

In a second step we can partially overcome this restriction and prove

### Proposition 0.2

Let  be a closed orientable hyperbolic manifold of dimension $n+1$ with $n\ne 2$ and let $\Gamma ^{\infty }(\mathrm {S}^2(T^*{\mathcal {M}}))$ be the space of smooth symmetric two-tensors endowed with its Fréchet topology and ${\mathscr {R}}_{{\mathcal {M}},<0}$ the open subset of Riemannian metrics of negative sectional curvature. Then there is an open neighborhood $U\subset {\mathscr {R}}_{{\mathcal {M}},<0}$ of  such that for all Riemannian metrics  one has0.1Here  is the geodesic vector field on the unit co-sphere bundle  with respect to .

Note that also in dimension $n+1=2$ we obtain the equality (0.1) only in a neighborhood of , whereas Dyatlov and Zworski prove the equality in this dimension for all . It seems thus reasonable to conjecture that the equality holds in all dimensions $n+1\ne 3$ for all , or at least for all  in those connected components of ${\mathscr {R}}_{\mathcal M,<0}$ that contain a metric of constant negative curvature.

We obtain Proposition 0.2 as a special case of a more general result on simultaneous perturbations of the Riemannian metric and the geodesic vector field. To state this result, consider in the situation of Proposition 0.2 some Riemannian metric  and an arbitrary Anosov vector field  on . Denoting by  the “perpendicular” subbundle formed by all co-vectors annihilating  fiber-wise, the multiplicities of the resonance zero of the Lie derivative  acting on sections of  and , respectively, are easy to relate under relatively mild assumptions: By Lemma 3.4 it suffices to assume that there is a one-form  on  with , , and  to have the relation0.2This is fulfilled, for example, if  is a contact form and  is a contact Anosov vector field with respect to . In particular, if  is the geodesic vector field, one can take  to be the canonical contact form given by the restriction of the Liouville one-form to . So (0.1) is in fact equivalent to the equation0.3In Sect. 3 we study the stability of the Eq. (0.3) upon simultaneous perturbations of the Riemannian metric and the geodesic vector field. We obtain the following main result:

### Theorem 0.3

If $\dim {{\mathcal {M}}}\ne 3$ and  is a metric of constant negative curvature, then there exists an open set $U\subset {\mathscr {R}}_{{\mathcal {M}},<0}$ containing  and a constant $\delta >0$ such that for all Riemannian metrics  and all vector fields  with^5^ one has the boundsand if there is a one-form  on  with  and , then the bounds improve to the equality

### Remark 0.4

If  is a contact form and  is contact with respect to , then, as mentioned above, the resonance multiplicities on the bundles  and  are related by (0.2). So Theorem 0.3 implies that the relations (0.1) and (0.3) remain valid for simultaneous small perturbations  of the metric  and small perturbations  of the geodesic vector field  within the class of contact vector fields.

We prove Theorem 0.3 by combining Proposition 0.1, which has been obtained by a quantum-classical correspondence, with the cohomology results of Dang-Rivière [DR19c] as well as some recent advances concerning the perturbation theory of Pollicott-Ruelle resonances [Bon18].

The main steps in the proof of Proposition 0.1, carried out in Sect. 2, can be roughly summarized as follows:

1. First we prove that $m_{{\mathcal {L}}_X, X^\perp }(0)=m_{{\mathcal {L}}_X, E_+^*}(0)$, i.e., every generalized resonant state *u* of the resonance zero actually lives only in the dual stable subbundle $E_+^*\subset X^\perp$.
2. Then we show that *u* lies in the kernel of the horocycle operator ${\mathcal {U}}_-$ (defined in Sect. 2.2), which means that it is a generalized *first band* resonant state. This is achieved by observing that ${\mathcal {U}}_- u$ is a generalized resonant state on the tensor bundle $E_+^*\otimes E_-^*\cong E_-^*\otimes E_-^*$. Decomposing ${\mathcal {U}}_- u$ into a symmetric and an antisymmetric part, we apply [DFG15] to show that the symmetric part must be zero and [Gai86] to show that the antisymmetric part must be zero.
3. By [KW19] there are no first band Jordan blocks, so it follows that *u* is actually a resonant state.
4. Since *u* is a first band resonant state, *u* corresponds to a distributional one-form $u_\infty$ on the sphere $S^n$, the boundary at infinity of the hyperbolic space ${\mathbb {H}}^{n+1}$. Then $u_\infty$ is invariant under a certain representation of the lattice $\Gamma \subset \mathrm {SO}(n+1,1)_0$ on the space of distributional one-forms on $S^n$, where ${{\mathcal {M}}}=\Gamma \backslash {\mathbb {H}}^{n+1}$.
5. We apply again Gaillard’s result [Gai86]; it says that $u_\infty$ is mapped by a Poisson transform to a harmonic one-form on ${{\mathcal {M}}}$ which is non-zero if *u* is non-zero and that all harmonic one-forms on ${{\mathcal {M}}}$ arise this way.

In Sect. 3 we then carry out the proof of Theorem 0.3 along roughly the following steps:

1. Using the “fiber-wise rescaling” diffeomorphism between the unit co-sphere bundles ,  with respect to two Riemannian metrics ,  on ${{\mathcal {M}}}$, we transfer the initial setup involving vector fields  to an equivalent setup involving vector fields  on the -independent space . This transfer is such that if  is close to  and  is close to the geodesic vector field , then  is close to . We choose  of constant negative curvature.
2. By applying Bonthonneau’s result [Bon18] on perturbations of Anosov vector fields to the transferred setup on , we obtain the inequality  for all  close enough to  and all vector fields  close enough to  (they are then automatically Anosov).
3. From the results of Dang-Rivière [DR19c] we get the lower bound $b_1({{\mathcal {M}}})\le$ for every negatively curved Riemannian metric  on ${{\mathcal {M}}}$ and every Anosov vector field  on , and this bound improves to $b_0({{\mathcal {M}}})+b_1({{\mathcal {M}}})$ if  preserves a non-closed one-form.
4. In the proof of Proposition 0.1 we observe  if $\dim {{\mathcal {M}}}\ne 3$.

## Pollicott-Ruelle Resonances for Geodesic Flows

### Anosov vector fields and perpendicular forms

Let  be a closed orientable Riemannian manifold of dimension $n+1$ with negative sectional curvature. Then the geodesic flow $\varphi _t$ on the unit co-sphere bundle $S^*{\mathcal {M}}$ is an Anosov flow which implies that there is a $d\varphi _t$-invariant Hölder continuous splitting of the tangent bundle $T(S^*{\mathcal {M}})$

1.1 $$\begin{aligned} T(S^*{\mathcal {M}}) = E_0\oplus E_+\oplus E_-, \end{aligned}$$

where $E_0 = {{\mathbb {R}}}X$ is the neutral bundle spanned by the geodesic vector field *X* and $E_{+}$, $E_{-}$ are the stable and unstable bundles, respectively (see e.g. [Kni02, p. 252]). Additionally, there is a smooth contact one-form $\alpha \in \Omega ^1(S^*{\mathcal {M}})$ which is simply the restriction of the Liouville one-form on $T^*{\mathcal {M}}$ to $S^*{\mathcal {M}}$. It fulfills

$$\begin{aligned} \iota _X\alpha =1,~~\ker (\alpha ) = E_+\oplus E_-,~~ d\alpha \text { is symplectic on } \ker (\alpha ),~~{\mathcal {L}}_X\alpha =0, \end{aligned}$$

where ${\mathcal {L}}_X$ denotes the Lie derivative. Note that the last two properties imply that $\alpha \wedge (d\alpha )^n$ is a nowhere-vanishing flow-invariant non-zero volume form which defines the Liouville measure on $S^*{\mathcal {M}}$. Using the contact one-form we get a splitting of the cotangent bundle into smooth subbundles

$$\begin{aligned} T^*(S^*{\mathcal {M}}) = {{\mathbb {R}}}\alpha \oplus X^\perp ,~~~X^\perp :=\{(x,\xi )\in T^*(S^*{\mathcal {M}}): \xi (X(x)) = 0\}. \end{aligned}$$

We will call the smooth sections of $X^\perp$ perpendicular one-forms and denote their space by $\Omega ^1_\perp (S^*{\mathcal {M}})$. More generally, we introduce for $p=0,\ldots n$ the space of perpendicular *p*-forms

$$\begin{aligned} \Omega ^p_\perp (S^*{\mathcal {M}}) := \{\omega \in \Omega ^p(S^*\mathcal M): \iota _X\omega =0 \}=\Gamma ^\infty (\Lambda ^p X^\perp ). \end{aligned}$$

By the Anosov splitting, the bundle $X^\perp$ can be further split into

1.2 $$\begin{aligned} X^\perp = E_+^* \oplus E_-^* , \end{aligned}$$

where the dual stable and unstable bundles are defined by $E_\pm ^*(E_0\oplus E_\mp )=0$. In contrast to the smoothness of $X^\perp$, the subbundles $E^*_\pm$ are only Hölder continuous unless ${\mathcal {M}}$ is a locally symmetric space of rank one.

More generally, we can consider an arbitrary Anosov vector field *Y* on $S^*{{\mathcal {M}}}$ (again, see e.g. [Kni02, p. 252] for the definition), for which we have a splitting of the form (1.1) with $E_0={{\mathbb {R}}}Y$ and we define the bundle

$$\begin{aligned} Y^\perp :=\{(x,\xi )\in T^*(S^*{\mathcal {M}}): \xi (Y(x)) = 0\}. \end{aligned}$$

#### Remark 1.1

(Complexifications) When addressing spectral questions involving an operator on any of the bundles mentioned so far, or on any subbundle of a tensor power of $T^*(S^*{{\mathcal {M}}})$, it is often more useful to work with the complexified bundle. For simplicity of notation we shall not explicitly distinguish in the following between real vector bundles and their complexifications. It will be clear from the context whether we refer to the real or the complexified bundle.

### Pollicott-Ruelle resonances on forms

Pollicott-Ruelle resonances were introduced by Pollicott [Pol85] and Ruelle [Rue86] in order to study mixing properties of hyperbolic flows (as mentioned before). In the last years it has been found out that these resonances can also be defined as poles of meromorphically continued resolvents (see [Liv04, BL07, GLP13, FS11, DZ16] for approaches using semiclassical analysis and [DG16, BW17] for generalizations to noncompact settings). We follow [DG16] to introduce the notion of Pollicott-Ruelle resonances on an arbitrary smooth complex vector bundle ${\mathcal {V}} \rightarrow S^*{\mathcal {M}}$. For a vector field $Y\in \Gamma ^\infty (T(S^*{{\mathcal {M}}}))$, a first order differential operator ${\mathbf {Y}}$ on ${\mathcal {V}}$ is called *admissible lift* of *Y* if

$$\begin{aligned} {\mathbf {Y}}(f{\mathbf {u}}) = (Yf){\mathbf {u}} + f{\mathbf {Y}} \mathbf{u},~~~~f\in \mathrm{C^{\infty }}(S^*{\mathcal {M}}), \;\mathbf{u}\in \Gamma ^\infty ({\mathcal {V}}). \end{aligned}$$

This applies in particular to the geodesic vector field *X*, admissible lifts of which will be denoted by ${{\mathbf {X}}}$. An example of an admissible lift of a vector field *Y* is the Lie derivative ${\mathcal {L}}_Y$ on any $d\varphi _t$-invariant subbundle of $\otimes ^p T^*(S^*{\mathcal {M}})$ for some $p\in {{\mathbb {N}}}_0$ (taking into account Remark 1.1), where $\varphi _t$ is the flow of *Y*. In Sect. 2 we will additionally consider covariant derivatives which are further examples of admissible lifts. After choosing a smooth metric on ${\mathcal {V}}$ one defines the space $\mathrm{L}^2(S^*{\mathcal {M}}, {\mathcal {V}})$. Note that by the compactness of ${\mathcal {M}}$ only the norm on this space depends on the choice of the metric but neither does the space nor its topology. Let now *Y* be an Anosov vector field on $S^*{\mathcal {M}}$ and ${\mathbf {Y}}$ an admissible lift as above. Then one checks [DG16, Eq. (1.10)] that there is a constant $C_{{\mathbf {Y}}}>0$ such that ${\mathbf {Y}} +\lambda :\mathrm{L}^2(S^*{\mathcal {M}}, {\mathcal {V}}) \rightarrow \mathrm{L}^2(S^*{\mathcal {M}}, {\mathcal {V}})$ is invertible for $\text {Re}(\lambda )>C_{{\mathbf {Y}}}$. The following statement was proved in the scalar case and for particular vector bundles in [FS11, DZ16, FT17] and is straightforward to adapt to the case of general vector bundles (see e.g. [DG16, Thm. 1]).

#### Proposition 1.2

The resolvent $R_{{\mathbf {Y}},{\mathcal {V}}}(\lambda ):=(\mathbf{Y}+\lambda )^{-1}: \mathrm{L}^2(S^*{\mathcal {M}},{{\mathcal {V}}})\rightarrow \mathrm{L}^2(S^*{\mathcal {M}},{{\mathcal {V}}})$, $\mathrm {Re}(\lambda )\gg 0$, has a continuation to the whole complex plane as a meromorphic family of bounded operators

$$\begin{aligned} R_{{\mathbf {Y}},{\mathcal {V}}}(\lambda ): \mathrm{C^{\infty }}(S^*{\mathcal {M}},{\mathcal {V}}) \rightarrow {\mathcal {D}}'(S^*{\mathcal {M}},{\mathcal {V}}). \end{aligned}$$

Moreover, for any pole $\lambda _0$ the residue operators $\Pi _{\lambda _0} = \mathrm{res} _{\lambda =\lambda _0}(R_{\mathbf {Y},{{\mathcal {V}}}}(\lambda ))$ have finite rank.

#### Definition 1.3

The poles of $R_{{\mathbf {Y}},{{\mathcal {V}}}}(\lambda )$ are called *Pollicott-Ruelle resonances* of ${\mathbf {Y}}$. Given a resonance $\lambda _0$, the finite-dimensional space ${{\mathrm {Res}}}_{{\mathbf {Y}}, \mathcal V}(\lambda _0):= \text {ran} (\Pi _{\lambda _0})\subset \mathcal D'(S^*{\mathcal {M}}, {\mathcal {V}})$ is the space of *generalized Pollicott-Ruelle resonant states* and we call $m_{{\mathbf {Y}},\mathcal V}(\lambda _0):= \dim _{{\mathbb {C}}}{{\mathrm {Res}}}_{{\mathbf {Y}}, {\mathcal {V}}}(\lambda _0)$ the *multiplicity* of the resonance $\lambda _0$.

If ${{\mathcal {V}}}=S^*{\mathcal {M}}\times {{\mathbb {C}}}$ is the trivial line bundle and ${\mathbf {Y}}=Y$, then we write just ${{\mathrm {Res}}}_{Y}(\lambda _0)$ and $m_{Y}(\lambda _0)$.

For any resonance $\lambda _0$ there exists a number $J(\lambda _0)\in {{\mathbb {N}}}$ such that the generalized resonant states have the following alternative description [DG16, Theorem 2]:

1.3 $$\begin{aligned} {{\mathrm {Res}}}_{{\mathbf {Y}},{\mathcal {V}}}(\lambda _0) =\{u\in \mathcal D'(S^*{\mathcal {M}},{\mathcal {V}}): ({\mathbf {Y}}+\lambda _0)^{J(\lambda _0)} u =0,~\text {WF} (u)\subset E_+^*\}. \end{aligned}$$

If $J(\lambda _0) =1$ we say that the resonance *has no Jordan block*. Otherwise, the space of *Pollicott-Ruelle resonant states* $\text {res}_{{\mathbf {Y}},{\mathcal {V}}}(\lambda _0) := \ker (\mathbf {Y}+\lambda _0)\cap {{\mathrm {Res}}}_{{\mathbf {Y}},{\mathcal {V}}}(\lambda _0)$ is a proper subspace of ${{\mathrm {Res}}}_{{\mathbf {Y}},{\mathcal {V}}}(\lambda _0)$.

Note that the co-sphere bundle $S^*{\mathcal {M}}$, the vector fields *Y* on it (in particular, the geodesic vector field *X*), as well as their resolvents, Pollicott-Ruelle resonances, and associated resonant states and multiplicities depend on the Riemannian metric . In Sect. 3 we will be interested in their variation under perturbations of . For this reason we will write , ,  in order to emphasize the dependence on . In the other sections we suppress the Riemannian metric in the notation.

## Multiplicities on Constant Curvature Manifolds

In this section we assume that  is a closed orientable hyperbolic^6^ manifold of dimension $n+1$.

### Proposition 2.1

If $$n\ne 2$$, then

$$\begin{aligned} m_{{\mathcal {L}}_X,X^\perp }(0) = b_1({\mathcal {M}}). \end{aligned}$$

Furthermore, the resonance zero has no Jordan block, and if $n\ge 3$, then zero is the unique leading resonance and there is a spectral gap.^7^

The first part of this result will be a central ingredient for Theorem 0.3. We will prove Proposition 2.1 using a quantum-classical correspondence. Such correspondences have recently been developed in various contexts (see [DFG15] for compact hyperbolic manifolds, [GHW18, Had18] for the convex co-compact setting, and [GHW20] for generalizations to general rank one manifolds). We will use the general framework for vector bundles developed by the authors in [KW19]. Additionally we use a Poisson transform due to Gaillard [Gai86] and combining both ingredients allows us to construct an explicit bijection between the Pollicott-Ruelle resonant states in perpendicular one forms and the kernel of the Hodge Laplacian.

### Remark 2.2

The dimension $n+1=3$ is an exception where the multiplicity is given by $m_{{\mathcal {L}}_X, X^\perp }(0)= 2b_1({\mathcal {M}})$. The deeper reason for this exception is that Gaillard’s Poisson transform is not bijective in this case. The exceptional case could also be treated with our methods by a more detailed analysis of Gaillard’s Poisson transform. This special case has however been worked out already in [DGRS19, Proposition 7.7] by factorizations of zeta functions, so we refrain from taking on the additional effort.

A crucial role in these quantum-classical correspondences is played by the so-called (generalized) first band resonant states

2.1 $$\begin{aligned} {{\mathrm {Res}}}^{\mathrm {1st}}_{{\mathbf {X}},{\mathcal {V}}}(\lambda _0) :={{\mathrm {Res}}}_{{\mathbf {X}},{\mathcal {V}}}(\lambda _0) \cap \ker \mathcal U_-,~~~~\text {res} ^{\mathrm {1st}}_{{\mathbf {X}},{\mathcal {V}}}(\lambda _0) :=\text {res}_{{\mathbf {X}},{\mathcal {V}}}(\lambda _0) \cap \ker {\mathcal {U}}_-, \end{aligned}$$

where ${\mathcal {U}}_-$ is the horocycle operator which we will introduce below in (2.14). Roughly speaking, first band resonant states are resonant states that are constant in the unstable directions. In the process of proving Proposition 2.1 we observe in Sect. 2.1 that in any dimension $n+1$, including $n+1=3$, one has

2.2 $$\begin{aligned} {{\mathrm {Res}}}^{\mathrm {1st}}_{\mathcal L_X,X^\perp }(0)={{\mathrm {Res}}}_{{\mathcal {L}}_X,X^\perp }(0), \end{aligned}$$

which means that all resonant states of the resonance zero are first band resonant states, even though for $n=1$ zero is not necessarily the leading resonance. Furthermore, we establish the following result:

### Proposition 2.3

On any closed orientable hyperbolic manifold ${\mathcal {M}}$ of dimension $n+1$ and for any $p=0,\ldots , n$ with $p\ne n/2$, one has

2.3 $$\begin{aligned} \dim _{{\mathbb {C}}}\mathrm {Res}^{\mathrm {1st}}_{{\mathcal {L}}_X,\Lambda ^p E^*_+}(0) =\dim _{{\mathbb {C}}}\mathrm {Res}^{\mathrm {1st}}_{\mathcal L_X,\Lambda ^p E^*_-}(-2p)=b_p({{\mathcal {M}}}). \end{aligned}$$

We consider this result to be of independent interest because it shows that also the higher Betti numbers can be recovered by considering Pollicott-Ruelle resonant states on certain vector bundles that are invariant under the horocycle transformation. Again the statement is obtained by constructing an explicit isomorphism onto the kernel of the Hodge Laplacian.

### Description of the geometry of ${{\mathcal {M}}}$ in Lie-theoretic terms

Any closed orientable connected hyperbolic manifold ${\mathcal {M}}$ of dimension $n+1$ can be written as a bi-quotient

$$\begin{aligned} {\mathcal {M}} =\Gamma \backslash {\mathbb {H}}^{n+1}= \Gamma \backslash G/ K, \end{aligned}$$

where $G=\mathrm {SO}(n+1,1)_0$,^8^$K\cong \mathrm {SO}(n+1)$, and $\Gamma \subset G$ is a cocompact torsion-free discrete subgroup. ${\mathcal {M}}$ is thus an example of a Riemannian locally symmetric space of rank one. There exists a very efficient Lie-theoretic language to describe the structure of ${\mathcal {M}}$, the co-sphere bundle $S^*{\mathcal {M}}$, as well as the invariant vector bundles which we introduce in this subsection. For more details we refer the reader to [GHW20, KW19] and for background information to the textbooks [Kna02, Hel01]. In the following we shall introduce the required abstract language in a quite concrete way, tailored to the particular group $G=\mathrm {SO}(n+1,1)_0$.

The Lie algebra $\mathbf{{\mathfrak {g}}}=\mathbf{{\mathfrak {so}}}(n+1,1)$ of *G* can be explicitly realized as a matrix algebra:

2.4 $$\begin{aligned} \mathbf{{\mathfrak {g}}}=\mathbf{{\mathfrak {so}}}(n+1,1)&=\Big \{\begin{pmatrix}k &{} p\\ p^T &{} 0 \end{pmatrix}:k\in \mathbf{{\mathfrak {so}}}(n+1),\; p\in {{\mathbb {R}}}^{n+1} \Big \}\nonumber \\&=\Big \{\begin{pmatrix}k &{} 0\\ 0 &{} 0 \end{pmatrix}:k\in \mathbf{{\mathfrak {so}}}(n+1) \Big \}\oplus \Big \{\begin{pmatrix}0 &{} p\\ p^T &{} 0 \end{pmatrix}:p\in {{\mathbb {R}}}^{n+1} \Big \}\nonumber \\&=:\mathbf{{\mathfrak {k}}}\oplus \mathbf{{\mathfrak {p}}}, \end{aligned}$$

where $\mathbf{{\mathfrak {so}}}(n+1)$ is the algebra of all real skew-symmetric matrices of size $n+1$. The involution $\theta :\mathbf{{\mathfrak {g}}}\rightarrow \mathbf{{\mathfrak {g}}}$ given by $\theta X=-X^T$, $X\in \mathbf{{\mathfrak {g}}}$, is called Cartan involution. The subspaces $\mathbf{{\mathfrak {k}}}$ and $\mathbf{{\mathfrak {p}}}$ are the eigenspaces of $\theta$ with respect to the eigenvalues 1 and $-1$, respectively. $\mathbf{{\mathfrak {k}}}$ is the Lie algebra of the group

$$\begin{aligned} K:=\exp (\mathbf{{\mathfrak {k}}})\subset G, \end{aligned}$$

where $\exp$ denotes the matrix exponential. We have $K\cong \mathrm {SO}(n+1)$. The splitting $\mathbf{{\mathfrak {g}}}=\mathbf{{\mathfrak {k}}}\oplus \mathbf{{\mathfrak {p}}}$ is called Cartan decomposition. This decomposition is $\mathrm {Ad}(K)$-invariant, where $\mathrm {Ad}(K)$ is the action of the matrix group *K* on the matrix algebra $\mathbf{{\mathfrak {k}}}$ by conjugation.

The tangent bundle $T{{\mathcal {M}}}=T(\Gamma \backslash G/K)$ can then be identified with the associated vector bundle $\Gamma \backslash G\times _{\mathrm {Ad}(K)} \mathbf{{\mathfrak {p}}}$, and similarly we identify $T^*{{\mathcal {M}}}= \Gamma \backslash G\times _{\mathrm {Ad}^*(K)} \mathbf{{\mathfrak {p}}}^*$, where $\mathrm {Ad}^*(K)$ is the dual representation of $\mathrm {Ad}(K)$.

Via the Killing form ${\mathfrak {B}}:\mathbf{{\mathfrak {g}}}\times \mathbf{{\mathfrak {g}}}\rightarrow {{\mathbb {R}}}$, which is given explicitly by ${\mathfrak {B}}(X,Y)=2n\, \mathrm {tr}(XY)$, and the Cartan involution $\theta$ we define an $\mathrm {Ad}(K)$-invariant inner product $\left\langle \cdot ,\cdot \right\rangle$ on $\mathbf{{\mathfrak {g}}}$ by

$$\begin{aligned} \left\langle X,Y \right\rangle :=-(2n)^{-1}{\mathfrak {B}}(X,\theta Y)=\mathrm {tr}(XY^T),\qquad X,Y\in \mathbf{{\mathfrak {g}}}. \end{aligned}$$

The restriction of $\left\langle \cdot ,\cdot \right\rangle$ to $\mathbf{{\mathfrak {p}}}\times \mathbf{{\mathfrak {p}}}$ then defines a Riemannian metric of constant curvature $-1$ on ${\mathcal {M}}$. We carry over the inner product to $\mathbf{{\mathfrak {g}}}^*$ using the isomorphism $\mathbf{{\mathfrak {g}}}\cong \mathbf{{\mathfrak {g}}}^*$ given by $X\mapsto \left\langle X,\cdot \right\rangle$.

We next want to describe the structure of the co-sphere bundle $S^*{\mathcal {M}}$ and the Anosov vector bundles $E_{0/+/-}$. To this end, we note that there is a maximal one-dimensional abelian subalgebra ${\mathfrak {a}} \subset \mathbf{{\mathfrak {p}}}$, given explicitly by

$$\begin{aligned} \mathbf{{\mathfrak {a}}}=\Big \{\begin{pmatrix}0 &{} p\\ p^T &{} 0 \end{pmatrix}: p^T=(0,\ldots ,0,H),H\in {{\mathbb {R}}}\Big \}\subset \mathbf{{\mathfrak {g}}}. \end{aligned}$$

We will denote the element in $\mathbf{{\mathfrak {a}}}$ for which $H=1$ in the description above by $H_0$ and we identify

$$\begin{aligned} \mathbf{{\mathfrak {a}}}\cong {{\mathbb {R}}}\end{aligned}$$

by mapping $H_0$ to 1. Defining subspaces $\mathbf{{\mathfrak {n}}}^\pm \subset \mathbf{{\mathfrak {g}}}$ by

2.5 $$\begin{aligned} \mathbf{{\mathfrak {n}}}^\pm :=\Bigg \{\begin{pmatrix}0 &{} v &{} \mp v\\ -v^T &{} 0 &{} 0\\ \mp v^T &{} 0 &{}0 \end{pmatrix}: v\in {{\mathbb {R}}}^n \Bigg \}, \end{aligned}$$

we see from (2.4) that one has two decompositions

$$\begin{aligned} \mathbf{{\mathfrak {g}}}=\mathbf{{\mathfrak {k}}}\oplus \mathbf{{\mathfrak {a}}}\oplus \mathbf{{\mathfrak {n}}}^+= \mathbf{{\mathfrak {k}}}\oplus \mathbf{{\mathfrak {a}}}\oplus \mathbf{{\mathfrak {n}}}^-. \end{aligned}$$

They are called Iwasawa decompositions. The spaces $\mathbf{{\mathfrak {n}}}^\pm$ are characterized by the property

2.6 $$\begin{aligned}{}[H_0,Y]=\pm Y\qquad \forall \; Y\in \mathbf{{\mathfrak {n}}}^\pm , \end{aligned}$$

and in fact they are the largest subspaces of $\mathbf{{\mathfrak {g}}}$ with these properties. In more abstract terms, the spaces $\mathbf{{\mathfrak {n}}}^\pm$ are the root spaces with respect to the roots $\pm \alpha _0$, where $\alpha _0\in \mathbf{{\mathfrak {a}}}^*$ is the element that maps $H_0$ to 1. We will identify

$$\begin{aligned} \mathbf{{\mathfrak {n}}}^\pm \cong {{\mathbb {R}}}^n \end{aligned}$$

by mapping each matrix as in (2.5) to the vector *v*. Also on the group level there are two corresponding Iwasawa decompositions $G=KAN^+= KAN^-.$ Here $N^\pm :=\exp (\mathbf{{\mathfrak {n}}}^\pm )\subset G$ and $A:=\exp (\mathbf{{\mathfrak {a}}})\subset G$ are the matrix subgroups with Lie algebras $\mathbf{{\mathfrak {n}}}^\pm$ and $\mathbf{{\mathfrak {a}}}$, respectively. For each group element $g\in G$ we now have unique Iwasawa ($+$) and opposite Iwasawa (−) decompositions

2.7 $$\begin{aligned} \begin{aligned} g&=k^+(g)a^+(g)n^+(g)=k^+(g)\exp (H^+(g))n^+(g)\\&=k^-(g)a^-(g)n^-(g)=k^-(g)\exp (H^-(g))n^-(g), \end{aligned} \end{aligned}$$

where $\exp (H^\pm (g))=a^\pm (g)$. In more concrete terms, this means that each matrix *g* in *G* can be written in a unique way as a product of three matrices in *K*, *A*, and $N^\pm$, respectively. Assigning to each matrix in *G* these unique matrices provides us with maps

2.8 $$\begin{aligned} k^\pm : G\rightarrow K,\qquad a^\pm : G\rightarrow A,\qquad H^\pm : G\rightarrow \mathbf{{\mathfrak {a}}},\qquad n^\pm : G\rightarrow N^\pm . \end{aligned}$$

In addition, we define the group

$$\begin{aligned} M:=\{m\in K:[m,a]=0\;\forall \; a\in A\}=\{m\in K:\mathrm {Ad}(m)(H)=0\;\forall \; H\in \mathbf{{\mathfrak {a}}}\}\subset K \end{aligned}$$

and let $\mathbf{{\mathfrak {m}}}$ be the Lie algebra of *M*. Explicitly, we have

$$\begin{aligned} \mathbf{{\mathfrak {m}}}=\Bigg \{\begin{pmatrix}m &{} 0 &{} 0\\ 0 &{} 0 &{} 0\\ 0 &{} 0 &{}0 \end{pmatrix}: m\in \mathbf{{\mathfrak {so}}}(n) \Bigg \}\subset \mathbf{{\mathfrak {k}}},\qquad M=\exp (\mathbf{{\mathfrak {m}}})\cong \mathrm {SO}(n). \end{aligned}$$

The groups $N^\pm$ are normalized by *A* and *M*. In fact, when identifying $\mathbf{{\mathfrak {n}}}^\pm \cong {{\mathbb {R}}}^n$ as above, then the $\mathrm {Ad}(M)$-action on $\mathbf{{\mathfrak {n}}}^\pm \cong {{\mathbb {R}}}^n$ is just the defining representation of $\mathrm {SO}(n)$ on ${{\mathbb {R}}}^n$. We have the so-called Bruhat decomposition

2.9 $$\begin{aligned} \mathbf{{\mathfrak {g}}}=\mathbf{{\mathfrak {m}}}\oplus \mathbf{{\mathfrak {a}}}\oplus \mathbf{{\mathfrak {n}}}^+\oplus \mathbf{{\mathfrak {n}}}^- \end{aligned}$$

which turns out to be invariant under the $\mathrm {Ad}(M)$-action.

The co-sphere bundle $S^*{\mathcal {M}}$ can be identified with $\Gamma \backslash G/M$. Indeed, the element $\alpha _0\in \mathbf{{\mathfrak {a}}}^*\subset \mathbf{{\mathfrak {p}}}^*$ introduced above fulfills $\left\| \alpha _0 \right\| =1$ and

$$\begin{aligned} \Gamma \backslash G/M\ni \Gamma gM\mapsto [\Gamma g,\alpha _0]\in S^*{\mathcal {M}}\subset T^*{{\mathcal {M}}}=\Gamma \backslash G\times _{\mathrm {Ad}^*(K)} \mathbf{{\mathfrak {p}}}^*\end{aligned}$$

is a well-defined diffeomorphism. The Lie group $A\cong {{\mathbb {R}}}$ acts from the right on $\Gamma \backslash G/M$ because it commutes by definition with *M*, and this action precisely coincides with the geodesic flow. In particular, the geodesic vector field $X\in \Gamma ^\infty (T(S^*{{\mathcal {M}}}))$ corresponds to the constant function ${\bar{X}}:G\rightarrow \mathbf{{\mathfrak {a}}}$ with ${\bar{X}}(g)=H_0$ for all $g\in G$. Furthermore, the tangent bundle of $S^*{\mathcal {M}}$ can be identified as follows:

2.10 $$\begin{aligned} T(S^*{\mathcal {M}})= & {} \Gamma \backslash G\times _{\mathrm {Ad}(M)} (\mathbf{{\mathfrak {a}}}\oplus \mathbf{{\mathfrak {n}}}^+ \oplus \mathbf{{\mathfrak {n}}}^-)\nonumber \\= & {} {{\mathbb {R}}}X\oplus \underbrace{\Gamma \backslash G\times _{\mathrm {Ad}(M)}\mathbf{{\mathfrak {n}}}^+}_{=E_+}\oplus \underbrace{\Gamma \backslash G\times _{\mathrm {Ad}(M)}\mathbf{{\mathfrak {n}}}^-}_{=E_-}. \end{aligned}$$

There is an analogous identification of $T^*(S^*{\mathcal {M}})$. The Anosov stable and unstable bundles $E_\pm$ can be described more concretely using their lifts ${\widetilde{E}}_\pm$ to the frame bundle $F{{\mathcal {M}}}=\Gamma \backslash G$ along the *M*-orbit projection $F{{\mathcal {M}}}=\Gamma \backslash G\rightarrow \Gamma \backslash G/M=S{{\mathcal {M}}}$: Choosing an orthonormal basis $U^\pm _1,\ldots ,U^\pm _n$ of $\mathbf{{\mathfrak {n}}}^\pm$, the constant function $G\rightarrow \mathbf{{\mathfrak {n}}}^\pm$ with value $U^\pm _j$ defines a nowhere-vanishing vector field on $F{\mathcal {M}}$, denoted also by $U^\pm _j$, and one has

2.11 $$\begin{aligned} \widetilde{E}_\pm =\mathrm {span}_{{{\mathbb {R}}}}(U^\pm _1,\ldots ,U^\pm _n). \end{aligned}$$

The boundary at infinity of the hyperbolic space ${\mathbb {H}}^{n+1}=G/K$ is diffeomorphic to the sphere $S^{n}$ and can be realized as

$$\begin{aligned} \partial _{\infty }{\mathbb {H}}^{n+1}=K/M=\mathrm {SO}(n+1)/\mathrm {SO}(n)\cong S^{n}. \end{aligned}$$

Consequently, the tangent bundle of $\partial _{\infty }{\mathbb {H}}^{n+1}$ can be identified with

$$\begin{aligned} T(K/M)=K\times _{\mathrm {Ad}(M)}\mathbf{{\mathfrak {m}}}^{\perp _\mathbf{{\mathfrak {k}}}}, \end{aligned}$$

where $\mathbf{{\mathfrak {m}}}^{\perp _\mathbf{{\mathfrak {k}}}}\subset \mathbf{{\mathfrak {k}}}$ denotes the orthogonal complement of $\mathbf{{\mathfrak {m}}}$ in $\mathbf{{\mathfrak {k}}}$, given explicitly by

$$\begin{aligned} \mathbf{{\mathfrak {m}}}^{\perp _\mathbf{{\mathfrak {k}}}}=\Bigg \{\begin{pmatrix}0 &{} v &{} 0\\ -v^T &{} 0 &{} 0\\ 0 &{} 0 &{}0 \end{pmatrix}: v\in {{\mathbb {R}}}^n \Bigg \}. \end{aligned}$$

We can identify $\mathbf{{\mathfrak {m}}}^{\perp _\mathbf{{\mathfrak {k}}}}\cong {{\mathbb {R}}}^n$ by mapping each matrix as above to *v*. The restriction of the representation $\mathrm {Ad}(M)$ to $\mathbf{{\mathfrak {m}}}^{\perp _\mathbf{{\mathfrak {k}}}}$ is then just the defining representation of $\mathrm {SO}(n)$ on ${{\mathbb {R}}}^n$.

In view of these identifications all vector bundles over $S^*{\mathcal {M}}$ of interest in the following are associated vector bundles of the form ${{\mathcal {V}}}_\tau :=G\times _\tau V$ with respect to some finite-dimensional complex *M*-representation $(\tau ,V)$.

As all our homogenous spaces are reductive there always exists a canonical connection that we denote by

2.12 $$\begin{aligned} \nabla : \Gamma ^\infty ({{\mathcal {V}}}_\tau )\rightarrow \Gamma ^\infty ({{\mathcal {V}}}_\tau \otimes T^*(S^*{\mathcal {M}})). \end{aligned}$$

To describe how $\nabla$ is defined, let us regard a section $s\in \Gamma ^\infty ({{\mathcal {V}}}_\tau )$ as a right-*M*-equivariant function $\bar{s}\in \mathrm{C^{\infty }}(\Gamma \backslash G,V)$. Moreover, by (2.10) we regard a vector field ${{\mathfrak {X}}}\in \Gamma ^\infty (T(S^*{\mathcal {M}}))$ as a right-*M*-equivariant function ${\bar{{{\mathfrak {X}}}}}\in \mathrm{C^{\infty }}(\Gamma \backslash G,\mathbf{{\mathfrak {n}}}^+\oplus \mathbf{{\mathfrak {a}}}\oplus {\mathbf{{\mathfrak {n}}}^-})$, that is, ${\bar{{{\mathfrak {X}}}}}(\Gamma gm)=\mathrm {Ad}(m^{-1}){\bar{{{\mathfrak {X}}}}}(\Gamma g)$ for every $m\in M$. Then $\nabla$ is defined by the covariant derivative

2.13 $$\begin{aligned} \begin{aligned} \nabla _{{{\mathfrak {X}}}}(s)(\Gamma gM):=\frac{d}{dt}\Big |_{t=0}{\bar{s}}\big (\Gamma ge^{t{\bar{{{\mathfrak {X}}}}}(\Gamma g)}\big ). \end{aligned} \end{aligned}$$

### Horocycle operators

Horocycle operators have been introduced in [DFG15] as a crucial tool for establishing quantum-classical correspondences. We already mentioned them in the definition of the first band resonant states (2.1) in the introduction. They are defined as follows: Let $({\mathcal {V}},\nabla )$ be a vector bundle over $S^*{{\mathcal {M}}}$ with a connection $\nabla$ and denote by $\widetilde{\text {pr}}_{E^*_-}:\Gamma ^\infty ({\mathcal {V}}\otimes T^*(S^*{{\mathcal {M}}}))\rightarrow \Gamma ^\infty ({\mathcal {V}}\otimes E^*_-)$ the map induced by the fiber-wise orthogonal projection $\text {pr}_{E^*_-}:T^*(S^*{\mathcal {M}}) \rightarrow E^*_-$ onto the subbundle $E^*_-\subset T^*(S^*{\mathcal {M}})$. Then we define the horocyle operator ${\mathcal {U}}_-$ of $({\mathcal {V}},\nabla )$ by composing the connection $\nabla : \Gamma ^\infty ({\mathcal {V}}) \rightarrow \Gamma ^\infty ({\mathcal {V}}\otimes T^*(S^*{{\mathcal {M}}}))$ with $\widetilde{\text {pr}}_{E^*_-}$:

2.14 $$\begin{aligned} {\mathcal {U}}_-:= \widetilde{\text {pr}}_{E^*_-} \circ \nabla : \Gamma ^\infty ({\mathcal {V}}) \rightarrow \Gamma ^\infty ({\mathcal {V}}\otimes E^*_-). \end{aligned}$$

By duality, ${\mathcal {U}}_-$ extends to distributional sections. In the concrete language of (2.11) we can express ${\mathcal {U}}_-$ as follows: If $\widetilde{\mathcal {V}}=\pi ^*{\mathcal {V}}$ is the lift of ${\mathcal {V}}$ to the frame bundle, i.e., the pullback bundle with respect to the *M*-orbit projection $\pi :F{{\mathcal {M}}}=\Gamma \backslash G\rightarrow \Gamma \backslash G/M=S{{\mathcal {M}}}$ and if ${\tilde{u}}\in \Gamma ^\infty (\widetilde{\mathcal {V}})$ is the lift of a section $u\in \Gamma ^\infty ({\mathcal {V}})$, then the lift of the section $\mathcal U_- u$ to the bundle $\widetilde{{\mathcal {V}}\otimes E^*_-}\cong \widetilde{\mathcal {V}}\otimes {\widetilde{E}}^*_-$ is given by

$$\begin{aligned} \widetilde{{\mathcal {U}}_- u}=\sum _{j=1}^n\widetilde{\nabla }_{U_j^-}{\tilde{u}}\otimes (U^-_j)^*, \end{aligned}$$

where $(U^-_j)^*\in \Gamma ^\infty ({\widetilde{E}}_-^*)$ is the dual vector field of $U^-_j$ and ${\widetilde{\nabla }}=\pi ^*\nabla$ the lifted (i.e., pullback) connection on ${\widetilde{{{\mathcal {V}}}}}$.

As already stated in (2.1), the so-called first band resonant states are defined as those resonant states that are annihilated by ${\mathcal {U}}_-$. The main technical feature of ${\mathcal {U}}_-$ is that it obeys the commutation relation

2.15 $$\begin{aligned} \nabla _X{\mathcal {U}}_-- {\mathcal {U}}_- \nabla _X =\mathcal U_-. \end{aligned}$$

This is a consequence of the commutation relations (2.6), the definition (2.13) of the covariant derivative, and the observation from Sect. 2.1 that the geodesic vector field *X* corresponds to the constant function ${\bar{X}}:G\rightarrow \mathbf{{\mathfrak {a}}}$ with value $H_0$. If $u\in {{\mathrm {Res}}}_{\nabla _X,{\mathcal {V}}}(\lambda )$ for some $\lambda \in {{\mathbb {C}}}$ and $J\in {{\mathbb {N}}}$ is such that $(\nabla _X+\lambda )^Ju=0$, then (2.15) implies

$$\begin{aligned} (\nabla _X+\lambda )^J\mathcal U_-u= & {} (\nabla _X+\lambda )^{J-1}(\nabla _X+\lambda )\mathcal U_-u\\= & {} (\nabla _X+\lambda )^{J-1}\mathcal U_-(\nabla _X+\lambda +1)u=\cdots ={\mathcal {U}}_-(\nabla _X+\lambda +1)^J u, \end{aligned}$$

which proves the following very useful *shifting property* of the horocycle operator ${\mathcal {U}}_-$:

2.16 $$\begin{aligned} \mathcal U_-\big (\mathrm {Res}_{\nabla _X,{\mathcal {V}}}(\lambda )\big )\subset {{\mathrm {Res}}}_{\nabla _X,{\mathcal {V}}\otimes E^*_-}(\lambda +1),\qquad {\mathcal {U}}_-\big (\mathrm {res}_{\nabla _X,\mathcal V}(\lambda )\big )\subset \mathrm {res}_{\nabla _X,{\mathcal {V}}\otimes E^*_-}(\lambda +1).\nonumber \\ \end{aligned}$$

### First band resonant states and principal series representations

As already mentioned above, the homogeneous space $K/M \cong S^{n}$ can be regarded as the *boundary at infinity* of the Riemannian symmetric space $G/K = {\mathbb {H}}^{n+1}$ and using the Iwasawa projection we can define a left-*G*-action

2.17 $$\begin{aligned} g(kM):=k^-(g k)M,\qquad g \in G,\;k\in K. \end{aligned}$$

Given a finite-dimensional complex *M*-representation $(\tau , V)$ we define the *boundary vector bundle*

$$\begin{aligned} {{{\mathcal {V}}}^{\mathcal {B}}_{\tau }} =(K\times _{\tau } V,\pi _{{{\mathcal {V}}}^{\mathcal {B}}_{\tau }}),\qquad \pi _{{{\mathcal {V}}}^{\mathcal {B}}_{\tau }}([k,v])=kM. \end{aligned}$$

The total space $K\times _{\tau } V$ of ${{\mathcal {V}}}^{\mathcal {B}}_{\tau }$ carries the *G*-action

2.18 $$\begin{aligned} g[k,v]:=[k^-(gk),v],\qquad g\in G,\;k\in K, \end{aligned}$$

that lifts the *G*-action (2.17) on the base space *K*/*M*. Consequently, we get an induced action on smooth sections:

2.19 $$\begin{aligned} (g s)(kM):={g}\big (s\big (g^{-1}(kM)\big )\big ),\qquad s\in \Gamma ^\infty ({{\mathcal {V}}}^{\mathcal {B}}_{\tau }),\;g\in G. \end{aligned}$$

If we consider a section $s\in \Gamma ^\infty ({{\mathcal {V}}}^{\mathcal {B}}_{\tau })$ as a right-*M*-equivariant smooth function ${\bar{s}}:K\rightarrow V$, the action (2.19) corresponds to assigning to $\bar{s}$ for any $g\in G$ the right-*M*-equivariant smooth function $\overline{g s}:K\rightarrow V$ given by

2.20 $$\begin{aligned} {\overline{gs}}(k)={\bar{s}}(k^-(g^{-1}k)),\qquad g\in G,\;k\in K. \end{aligned}$$

To describe how the principal series representation of *G* associated to an *M*-representation $\tau$ and a parameter $\lambda \in {{\mathbb {C}}}$ acts on smooth sections of ${\mathcal {V}}_\tau ^{{\mathcal {B}}}$, let us regard a section $s\in \Gamma ^\infty (\mathcal V_\tau ^{{\mathcal {B}}})$ as a right-*M*-equivariant function ${\bar{s}}\in \mathrm{C^{\infty }}(K,V)$. We then set^9^

2.21 $$\begin{aligned} \overline{\pi ^{\lambda }_{\tau }(g)s}(k):=e^{(\lambda + n/2)H^-(g^{-1}k)}{\bar{s}}(k^{-}(g^{-1}k)),\quad s\in \Gamma ^\infty ({{\mathcal {V}}}^{{\mathcal {B}}}_\tau ),\; kM\in K/M.\qquad \end{aligned}$$

This representation extends by continuity to a representation $\pi ^{\lambda }_{\tau }:G\rightarrow \mathrm {End}({{\mathcal {D}}}'(K/M,{{\mathcal {V}}}^{\mathcal {B}}_{\tau }))$. One has the following important relation between first band resonant states and the $\Gamma$-invariant distributional sections of the boundary vector bundle with respect to the principal series representation $\pi ^{-\lambda -n/2}_\tau$.

#### Proposition 2.4

( [KW19, Lemma 2.15]) For each $\lambda \in {{\mathbb {C}}}$ there is an explicit isomorphism

2.22 $$\begin{aligned} Q_{\lambda }:\mathrm{res}^{\mathrm {1st}}_{\nabla _X,{{\mathcal {V}}}_{\tau }}(\lambda ){\mathop {\longrightarrow }\limits ^{\cong }}{^\Gamma }\big ({{\mathcal {D}}}'(K/M,{{\mathcal {V}}}^{\mathcal {B}}_{\tau }), \pi ^{-\lambda -n/2}_\tau \big ) \end{aligned}$$

onto the space of all distributional sections *u* of ${{\mathcal {V}}}^{\mathcal {B}}_{\tau }$ with $\pi ^{-\lambda -n/2}_\tau (\gamma )u=u$ for every $\gamma \in \Gamma$.

### Relating resonances of the Lie- and covariant derivatives

Proposition 2.4 provides a powerful way to handle first band resonant states of the covariant derivative $\nabla _X$ along the geodesic vector field. In Propositions 2.1 and 2.3 we are however interested in resonant states of the Lie derivative. Therefore we have to relate these states:

#### Lemma 2.5

For $p\in \{0,1,2,\ldots \}$, suppose that $\tau$ is a subrepresentation of $\otimes ^p(\mathrm {Ad}(M)|_{\mathbf{{\mathfrak {n}}}^\pm })$. Then the covariant derivative and the Lie derivative along the geodesic vector field *X*, acting on smooth sections of ${{\mathcal {V}}}_{\tau }$, are related by

$$\begin{aligned} \mathcal {L}_{X} = \nabla _{X} \mp p\,\mathrm {id}_{\Gamma ^\infty ({{\mathcal {V}}}_{\tau })}. \end{aligned}$$

Consequently, one has for every $\lambda \in {{\mathbb {C}}}$ and $p\in {{\mathbb {N}}}$

2.23 $$\begin{aligned} \mathrm {Res}_{{\mathcal {L}}_X,{{\mathcal {V}}}_\tau }(\lambda )=\mathrm {Res}_{\nabla _X, {{\mathcal {V}}}_\tau }(\lambda \mp p) ~~ \mathrm{and } ~~ \mathrm {res}_{\mathcal L_X,{{\mathcal {V}}}_\tau }(\lambda )=\mathrm {res}_{\nabla _X, {{\mathcal {V}}}_\tau }(\lambda \mp p). \end{aligned}$$

#### Proof

Recall that the geodesic flow on $S^*(\Gamma \backslash G/K)=\Gamma \backslash G/M$ is given by

2.24 $$\begin{aligned} \varphi _t(\Gamma gM)=\Gamma ge^{tH_0}M,\qquad t\in {{\mathbb {R}}}. \end{aligned}$$

Its derivative $d\varphi _t:T(\Gamma \backslash G/M)=\Gamma \backslash G\times _{\mathrm {Ad}(M)}(\mathbf{{\mathfrak {n}}}^+\oplus \mathbf{{\mathfrak {a}}}\oplus \mathbf{{\mathfrak {n}}}^-)\rightarrow \Gamma \backslash G\times _{\mathrm {Ad}(M)}(\mathbf{{\mathfrak {n}}}^+\oplus \mathbf{{\mathfrak {a}}}\oplus \mathbf{{\mathfrak {n}}}^-)$ reads

2.25 $$\begin{aligned} d\varphi _t(\Gamma gM)([\Gamma g M, v])= & {} [\Gamma g M, \mathrm {Ad}(e^{-tH_0})v]\nonumber \\&\in \Gamma \backslash G\times _{\mathrm {Ad}(M)}(\mathbf {{\mathfrak {n}}}^+\oplus \mathbf {{\mathfrak {a}}}\oplus \mathbf {{\mathfrak {n}}}^-),\qquad t\in {{\mathbb {R}}},\;[\Gamma g M, v]. \nonumber \\ \end{aligned}$$

Any vector $v\in \mathbf{{\mathfrak {n}}}^\pm$ is an eigenvector of the adjoint action:

2.26 $$\begin{aligned} \mathrm {Ad}(e^{-tH_0})v =e^{-t\mathrm {ad}(H_0)}v =e^{\mp t} v. \end{aligned}$$

Let now $\omega \in \Gamma ^\infty ({{\mathcal {V}}}_\tau )$, identified with a left-$\Gamma$-, right-*M*-equivariant function ${\overline{\omega }}: G\rightarrow V$, where $V\subset \otimes ^p (\mathbf{{\mathfrak {n}}}^\pm )$. Considering $\varphi _t$ as a left-$\Gamma$-, right-*M*-equivariant map $\bar{\varphi }_t: G\rightarrow G$, let $\overline{\varphi _t^*\omega }: G\rightarrow V$ be the left-$\Gamma$-, right-*M*-equivariant function corresponding to $\varphi _t^*\omega \in \Gamma ^\infty ({{\mathcal {V}}}_\tau )$. Then we get with (2.26) for $g\in G$ and $v_1,\ldots ,v_p\in \mathbf{{\mathfrak {n}}}^\pm$:

$$\begin{aligned} \overline{\varphi _t^*\omega }(g)(v_1,\ldots ,v_p) =\bar{\omega }(ge^{tH_0})(e^{\mp t }v_1,\ldots ,e^{\mp t }v_p)=e^{\mp p t}{\bar{\omega }}(ge^{tH_0})(v_1,\ldots ,v_p). \end{aligned}$$

For the Lie derivative of $\omega$ we then obtain with the analogous “$\,\bar{\;}\bar{\;}\,$”-notation and the product rule

$$\begin{aligned} \overline{\mathcal {L}_{X}\omega }(g)(v_1,\ldots ,v_p)&=\frac{d}{dt}\Big |_{t=0}\overline{\varphi _t^*\omega }(g)(v_1,\ldots ,v_p)\\&=\frac{d}{dt}\Big |_{t=0}\Big (e^{\mp p t }{\bar{\omega }}(ge^{tH_0})(v_1,\ldots ,v_p)\Big )\\&=\frac{d}{dt}\Big |_{t=0}{\bar{\omega }}(ge^{tH_0})(v_1,\ldots ,v_p) \mp p {\bar{\omega }}(g)(v_1,\ldots ,v_p) \\&=\overline{\nabla _{X}\omega }(g)(v_1,\ldots ,v_p) \mp p {\bar{\omega }}(g)(v_1,\ldots ,v_p). \end{aligned}$$

Here we recalled the definition (2.13) of the canonical covariant derivative. $\square$

### Proof of Proposition 2.3

Let us collect what we have obtained so far: By Lemma 2.5

$$\begin{aligned} \mathrm {res}^{\mathrm {1st}}_{{\mathcal {L}}_X,\Lambda ^p E^*_+}(0) = \mathrm {res}^{\mathrm {1st}}_{\nabla _X,\Lambda ^p E^*_+}(-p) ~~ and ~~ \mathrm {res}^{\mathrm {1st}}_{{\mathcal {L}}_X,\Lambda ^p E^*_-}(-2p) = \mathrm {res}^{\mathrm {1st}}_{\nabla _X,\Lambda ^p E^*_-}(-p). \end{aligned}$$

As the adjoint action of *M* on $\mathbf{{\mathfrak {n}}}^\pm$ is given by the defining representation of $\mathrm {SO}(n)$ on ${{\mathbb {R}}}^n$ we deduce from (2.10) that $\Lambda ^p(E^*_\pm ) = \Gamma \backslash G\times _{\tau _p} \Lambda ^p({{\mathbb {R}}}^n)$ with $\tau _p$ being the p-th exterior power of the standard action of $\mathrm {SO}(n)$ on ${{\mathbb {R}}}^n$. By Proposition 2.4 we can thus identify

$$\begin{aligned} \mathrm {res}^{\mathrm {1st}}_{{\mathcal {L}}_X,\Lambda ^p E^*_+}(0) \cong \mathrm {res}^{\mathrm {1st}}_{{\mathcal {L}}_X,\Lambda ^p E^*_-}(-2p) \cong {^\Gamma }\big ({{\mathcal {D}}}'(K/M,{{\mathcal {V}}}^{\mathcal {B}}_{\tau _p}), \pi ^{p-n/2}_{\tau _p}\big ). \end{aligned}$$

We now use a vector-valued Poisson transform. To this end, let $\Delta _H = d\delta + \delta d$ be the Hodge Laplacian on $\Omega ^p({\mathbb {H}}^{n+1})$.

#### Theorem 2.6

(Poisson transform for $\Gamma$-invariant *p*-forms) Let $K=\mathrm {SO}(n+1)$, $M=\mathrm {SO}(n)$, and let $\tau _p$ be the *p*-th exterior power of the defining representation of $\mathrm {SO}(n)$ on ${{\mathbb {R}}}^n$. Then for any $\lambda \in {{\mathbb {C}}}$ with $\lambda \ne n-p$ and $\lambda \ne n+1, n+2,\ldots$, there is an isomorphism of vector spaces

$$\begin{aligned} P_{\tau _p,\lambda }&: {^\Gamma }\big ({{\mathcal {D}}}'(K/M,{{\mathcal {V}}}^{\mathcal {B}}_{\tau _p}), \pi ^{\lambda -n/2}_{\tau _p}\big ) \rightarrow \big \{\omega \in \Omega ^p({{\mathcal {M}}}): \\&\quad \Delta _H \omega = (\lambda -p)(n-\lambda - p)\omega ,~\delta \omega =0\big \}. \end{aligned}$$

This result is due to Gaillard (see [Gai86, Thm. 2’ c) and Thm. 3’], taking into account that $\Gamma$-invariant smooth forms are trivially *slowly growing* in Gaillard’s sense because $\Gamma$ is co-compact) although it requires some work (see Sect. 2.7) to translate his statements into the form stated above that we can apply in our setting. For $p\ne n/2$ the Poisson transform $P_{\tau _p,p}$ is bijective and thus

$$\begin{aligned} {^\Gamma }\big ({{\mathcal {D}}}'(K/M,{{\mathcal {V}}}^{\mathcal {B}}_{\tau _p}), \pi ^{p-n/2}_{\tau _p}\big ) \cong \left\{ \omega \in \Omega ^p({{\mathcal {M}}}), \Delta _H\omega =0,\delta \omega =0\right\} . \end{aligned}$$

As on compact manifolds any harmonic form is co-closed, the right hand side is simply the kernel of the Hodge Laplacian and Hodge theory implies that its dimension equals the *p*-th Betti number of ${{\mathcal {M}}}$. We thus have shown

$$\begin{aligned} \dim \mathrm {res}^{\mathrm {1st}}_{\nabla _X,\Lambda ^p E^*_+}(-p) = \dim \mathrm {res}^{\mathrm {1st}}_{{\mathcal {L}}_X,\Lambda ^p E^*_-}(-2p) = b_p({\mathcal {M}}). \end{aligned}$$

Now using once more that $p\ne n/2$ [KW19, Theorem 6.2] implies that the resonance at $-p$ of $\nabla _X$ has no Jordan block and consequently

2.27 $$\begin{aligned} \dim \mathrm {Res}^{\mathrm {1st}}_{{\mathcal {L}}_X,\Lambda ^p E^*_+}(0)= & {} \dim \mathrm {Res}^{\mathrm {1st}}_{\mathcal L_X,\Lambda ^p E^*_-}(-2p)= \dim \mathrm {Res}^{\mathrm {1st}}_{\nabla _X,\Lambda ^p E^*_+}(-p)\nonumber \\= & {} \dim \mathrm {res}^{\mathrm {1st}}_{\nabla _X,\Lambda ^p E^*_+}(-p) =b_p({{\mathcal {M}}}). \end{aligned}$$

This finishes the proof of Proposition 2.3.

### Proof of Proposition 2.1

Let $\lambda \in {{\mathbb {C}}}$. By the decomposition (1.2) and Lemma 2.5, we have

$$\begin{aligned} {{\mathrm {Res}}}_{{\mathcal {L}}_X,X^\perp }(\lambda )\cong \mathrm {Res}_{\mathcal L_X,E^*_+}(\lambda )\oplus \mathrm {Res}_{\mathcal L_X,E^*_-}(\lambda )=\mathrm {Res}_{\nabla _X, E^*_+}(\lambda -1)\oplus \mathrm {Res}_{\nabla _X,E^*_-}(\lambda +1). \end{aligned}$$

As $\nabla _X$ is an antisymmetric operator in $\mathrm{L}^2(E^*_-)$ there are no resonances of $\nabla _X$ on $E_-$ with positive real part^10^, so if $\mathrm {Re}\,\lambda >-1$ one has

2.28 $$\begin{aligned} {{\,\mathrm{Res}\,}}_{{\mathcal {L}}_X,X^\perp }(\lambda )\cong \mathrm {Res}_{\nabla _X, E^*_+}(\lambda -1). \end{aligned}$$

By the definition of first band resonant states (2.1) and the dimension formula for linear maps we conclude

2.29 $$\begin{aligned} \dim {{\mathrm {Res}}}_{\nabla _X, E^*_+}(\lambda -1) = \dim {\mathrm {Res}}^{\mathrm {1st}}_{\nabla _X, E^*_+}(\lambda -1) + \dim \mathcal {U}_-\big ({{\mathrm {Res}}}_{\nabla _X, E^*_+}(\lambda -1)\big ).\quad \end{aligned}$$

Regarding the statement on the leading resonance, we note that if $n\ge 3$ and $\mathrm {Re}\,\lambda >-1$, then by Proposition 2.4 and Theorem 2.6 there is an isomorphism

2.30 $$\begin{aligned} \mathrm {res}^{\mathrm {1st}}_{\nabla _X,E^*_+}(\lambda -1) \cong \{\omega \in \Gamma ^\infty (T^*{{\mathcal {M}}}): \Delta _H\omega =-\lambda (n+\lambda - 2)\omega , ~\delta \omega =0\}, \end{aligned}$$

where $\Delta _H$ is the Hodge Laplacian on ${{\mathcal {M}}}$. When $\mathrm {Re}\,\lambda > 1-\frac{n}{2}$, the eigenvalue $-\lambda (n+\lambda - 2)$ is real and positive iff $\lambda \in (1-\frac{n}{2},0]$ and if this does not hold the right hand side of (2.30) is the zero space. It follows for $n\ge 3$ and $\mathrm {Re}\,\lambda > 1-\frac{n}{2}$ that ${\mathrm {Res}}^{\mathrm {1st}}_{\nabla _X, E^*_+}(\lambda -1)=\{0\}$ unless $\lambda \in (1-\frac{n}{2},0]$ because every Jordan block would contain at least one resonant state. Now, in view of Proposition 2.3, (2.27), and (2.29), it remains to prove $\mathcal {U}_-({{\mathrm {Res}}}_{\nabla _X, E^*_+}(\lambda -1)) = 0$ under the assumption that $n \ne 2$ and $\mathrm {Re}\,\lambda > -\delta$ for some small $\delta >0$ to establish Proposition 2.1. Recall from (2.14) that $\mathcal {U}_-({{\mathrm {Res}}}_{\nabla _X, E^*_+}(\lambda -1)) \subset {\mathcal {D}}'({\mathcal {M}}, E^*_+\otimes E^ *_-)$. Further, by (2.16) one has

$$\begin{aligned} \mathcal {U}_-\big ({{\mathrm {Res}}}_{\nabla _X, E^*_+}(\lambda -1)\big ) \subset {{\mathrm {Res}}}_{\nabla _X, E^*_+\otimes E^ *_-}(\lambda ). \end{aligned}$$

If $\mathrm {Re}\,\lambda >0$, we immediately get the zero space on the right hand side as otherwise there would be resonances of $\nabla _X$ with positive real part, which is impossible by the antisymmetry of $\nabla _X$ in $\mathrm{L}^2(E^*_+\otimes E^ *_-)$, cf. Footnote 10. We are left with the proof of $\mathcal {U}_-({{\mathrm {Res}}}_{\nabla _X, E^*_+}(\lambda -1)) = 0$ for $\mathrm {Re}\,\lambda \in (-\delta ,0]$ with some small $\delta >0$. Another application of (2.16) and the absence of resonances of $\nabla _X$ with positive real part due to antisymmetry implies

$$\begin{aligned} {{\mathrm {Res}}}_{\nabla _X, E^*_+\otimes E^ *_-}(\lambda ) = {{\mathrm {Res}}}^ {\mathrm {1st}}_{\nabla _X, E^*_+\otimes E^ *_-}(\lambda )\qquad \text{ if } \mathrm {Re}\,\lambda > -1. \end{aligned}$$

Using the quantum-classical correspondence once more we shall obtain a simple description of the latter spaces. To this end, note that the Cartan involution $\theta |_{\mathbf{{\mathfrak {n}}}^+}:\mathbf{{\mathfrak {n}}}^+\rightarrow \mathbf{{\mathfrak {n}}}^-$ is an equivalence of representations $\mathrm {Ad}(M)|_{\mathbf{{\mathfrak {n}}}^+}\sim \mathrm {Ad}(M)|_{\mathbf{{\mathfrak {n}}}^-}$ which induces an isomorphism $E^*_+\cong E^*_-$ that is compatible with the connections on the two bundles. This in turn induces a connection-compatible isomorphism $E^*_+\otimes E^*_-\cong E^*_-\otimes E^*_-$. As the covariant derivatives $\nabla _X$ as well as the horocycle operators ${\mathcal {U}}_-$ are defined in terms of the respective connections, we conclude

$$\begin{aligned} {{\mathrm {Res}}}^{\mathrm {1st}}_{\nabla _X, E^*_+\otimes E^*_-}(\lambda )\cong {{\mathrm {Res}}}^{\mathrm {1st}}_{\nabla _X, E^*_-\otimes E^*_-}(\lambda ). \end{aligned}$$

Now let  be the Riemannian metric on $S^*{\mathcal {M}}$ induced by the Sasaki metric on $T^*{{\mathcal {M}}}$ with respect to the Riemannian metric on ${{\mathcal {M}}}$. The restriction of  to $E_-\times E_-$ defines a smooth section of $E^*_-\otimes E^*_-$.

If $n=2$, then $\Lambda ^2E^*_-\subset E^*_-\otimes E^*_-$ is the top-degree exterior power of $E_-$ and hence trivialized by choosing an orientation form $\Omega _{E_-}$ on $E_-$. Choosing a non-zero element $\Omega _0\in \Lambda ^2(\mathbf {{\mathfrak {n}^-}})^*$, we can define $\Omega _{E_-}$ to be the smooth section of $\Lambda ^2E^*_-=\Gamma \backslash G\times _{\Lambda ^2 \mathrm {Ad}^*(M)}\Lambda ^2(\mathbf {{\mathfrak {n}}}^-)^*$ induced by the constant function $G\rightarrow \Lambda ^2(\mathbf {{\mathfrak {n}}}^-)^*$ with the value $\Omega _0$.

#### Lemma 2.7

There is a number $\delta >0$ such that for all $\lambda \in {{\mathbb {C}}}$ with $\mathrm {Re}\,\lambda \in (-\delta ,0]$ one has

Before proving this lemma let us see how it finishes the proof of Proposition 2.1 and (2.2): All that is left to prove is that if ${\mathcal {U}}_- s=c \eta$ with , $s\in {{\,\mathrm {Res}}}_{\nabla _X, E^*_+}(-1)$, and $c\in {{\mathbb {C}}}$, then $c=0$. This is easy:^11^ If ${\mathcal {U}}_- s=c \eta$, then

$$\begin{aligned} \big <{\mathcal {U}}_- s,\eta \big >_{\mathrm {L}^2(S^*{{\mathcal {M}}},E^*_-\otimes E^*_-)}=c \Vert \eta \Vert _{\mathrm {L}^2(S^*{{\mathcal {M}}},E^*_-\otimes E^*_-)}^2. \end{aligned}$$

Thus, if ${\mathcal {U}}^*_-$ is the formal adjoint of $\mathcal U_-$, we have

2.31 $$\begin{aligned} s({\mathcal {U}}^*_-(\eta ))=c \Vert \eta \Vert _{\mathrm {L}^2(S^*{{\mathcal {M}}},E^*_-\otimes E^*_-)}^2, \end{aligned}$$

where the left hand side is the pairing of the distributional section *s* with the smooth section ${\mathcal {U}}^*_-(\eta )$. In [DFG15, Lemma 4.3] it is shown that ${\mathcal {U}}^*_-=-\mathcal T\circ {\mathcal {U}}_-$, ${\mathcal {T}}$ being the trace operator. The smooth section $\eta$ vanishes under all covariant derivatives as it corresponds to the constant function $G\rightarrow (\mathbf {{\mathfrak {n}}}^-)^*\otimes (\mathbf {{\mathfrak {n}}}^-)^*$ with either the value $\left\langle \cdot ,\cdot \right\rangle |_{\mathbf {{\mathfrak {n}}}^-\times \mathbf {{\mathfrak {n}}}^-}$ or the value $\Omega _0$. Therefore, we find ${\mathcal {U}}^*_-(\eta )=0$ and (2.31) implies $c=0$.

It remains to prove Lemma 2.7:

#### Proof of Lemma 2.7

The tensor product $E^*_-\otimes E^*_-$ splits into a sum of three subbundles according towhere $S^2_0(E^*_-)$ denotes the trace-free symmetric tensors of rank 2. Note that  is a trivial line bundle and for $n=1$ the other two bundles have rank zero. By the additivity of resonance multiplicities with respect to Whitney sums of vector bundles, we arrive at2.32Now we can consider the three summands on the right hand side individually. According to [DFG15, Lemmas 4.7 and 5.6, Thm. 6], there is for $\mathrm {Re}\,\lambda >-1$ an isomorphism

2.33 $$\begin{aligned} \text {res} ^{\mathrm {1st}}_{\nabla _X,S^2_0(E^*_-)}(\lambda )\cong \{\omega \in \Gamma ^\infty (S^2_0(T^*{{\mathcal {M}}})):\Delta _B\omega =-\lambda (n+\lambda )+2, ~\mathrm {div}\,\omega =0\},\nonumber \\ \end{aligned}$$

where $\Delta _B$ is the Bochner Laplacian associated to the connection $\nabla$. The eigenvalue $-\lambda (n+\lambda )+2$ appearing here is a real number iff $\mathrm {Im}\,\lambda =0$ or $\mathrm {Re}\,\lambda =-\frac{n}{2}$, so for $\mathrm {Re}\,\lambda >-\frac{1}{2}$ only numbers $\lambda \in (-1/2,\infty )$ remain as possible candidates for a non-zero resonance space (2.33). In addition, a Weitzenböck type formula (see [DFG15, Lemma 6.1]) says that the spectrum of $\Delta _B$ acting on $\Gamma ^\infty (S^2_0(T^*{{\mathcal {M}}}))$ is bounded from below by $n+1$ which is strictly larger than $-\lambda (n+\lambda )+2$ for $n\ge 2$ and $\lambda \in (-1/2,\infty )$. Consequently, for such *n* and $\lambda$ the right hand side of (2.33) is the zero space and it follows that ${{\mathrm {Res}}}^{\mathrm {1st}}_{\nabla _X,S^2_0(E^*_-)}(\lambda )=\{0\}$ because every Jordan block would contain at least one resonant state. Turning to the second summand in (2.32), we apply once more Proposition 2.4 and Theorem 2.6 and obtain for $n\ne 2$ an isomorphism

2.34 $$\begin{aligned} \mathrm {res}^{\mathrm {1st}}_{\nabla _X,\Lambda ^2E^*_-}(\lambda )\cong \{\omega \in \Gamma ^\infty (\Lambda ^2(T^*{{\mathcal {M}}})):\Delta _H\omega =-(\lambda +2)(n+\lambda -2), ~\delta \omega =0\}. \nonumber \\ \end{aligned}$$

For $\mathrm {Re}\,\lambda >-1$ and $n\ge 3$, the eigenvalue appearing here is either imaginary or negative, so the right hand side of (2.34) is the zero space (because $\Delta _H$ is positive) and $\mathrm {res}^{\mathrm {1st}}_{\nabla _X,\Lambda ^2E^*_-}(\lambda )=\{0\}$, ${{\mathrm {Res}}}^{\mathrm {1st}}_{\nabla _X,\Lambda ^2E^*_-}(\lambda )=\{0\}$.

When $n=2$ we have $\Lambda ^2E^*_-={{\mathbb {R}}}\Omega _{E_-}$. We can thus treat the second summand in (2.32) for $n=2$ and the third summand in (2.32) for arbitrary *n* in the same way: As $\nabla _X^J ({\tilde{c}}\Omega _{E_-})=(X^J\tilde{c})\,\Omega _{E_-}$ and  for each $J\in {{\mathbb {N}}}$, we see that the distributions $c,{\tilde{c}}$ have to be generalized scalar resonant states of a resonance $\lambda$. In the scalar case we can however apply Liverani’s result on the spectral gap for contact Anosov flows [Liv04] to see that zero is the unique leading resonance, with (generalized) resonant states the locally constant functions, and there is a spectral gap $\delta >0$, so the proof is finished. $\square$

### Gaillard’s Poisson transform

In his article [Gai86] Gaillard considers the vector-valued Poisson transform to which we refer in Theorem 2.6 in the special case of $\Gamma$-invariant elements. His notation and conventions are however quite different from ours. In the following we will translate his results into the form stated in Theorem 2.6.

Gaillard proves in [Gai86, Therems 2’, 3’] that *slowly growing* co-closed *p*-forms on ${\mathbb {H}}^{n+1}$ in appropriate eigenspaces of the Hodge Laplacian on ${\mathbb {H}}^{n+1}$ are the Poisson transforms of *p*-currents on *K*/*M*. When considering only *p*-forms on ${\mathbb {H}}^{n+1}$ that are $\Gamma$-invariant with respect to the action of $\Gamma$ by pullbacks, which we identify with *p*-forms on the compact quotient ${{\mathcal {M}}}=\Gamma \backslash {\mathbb {H}}^{n+1}$ in Theorem 2.6, the slow growth condition becomes redundant. The remaining task is to relate Gaillard’s pullback *G*-actions on *p*-currents to our principal series representations of *G* on distributional sections.

We will denote the space of *p*-currents on *K*/*M* by $\mathcal D_p'(K/M):=(\Omega ^{n-p}(K/M))'$, and we have the canonical dense embedding . As *G* acts by diffeomorphisms on *K*/*M* the pullback action on ${\mathcal {D}}'_p(K/M)$ provides a *G*-representation.

#### Lemma 2.8

The pullback action of *G* on the space ${\mathcal {D}}'_p(K/M)$ of *p*-currents is equivalent to the principal series representation $\pi ^{p-n/2}_{\tau _p}$ on ${{\mathcal {D}}}'(K/M,{\mathcal {V}}^{\mathcal B}_{\tau _p})$.

#### Proof

Denote by $\mathbf{{\mathfrak {m}}}^{\perp _\mathbf{{\mathfrak {k}}}}\subset \mathbf{{\mathfrak {k}}}$ the orthogonal complement of $\mathbf{{\mathfrak {m}}}$ in $\mathbf{{\mathfrak {k}}}$. Then *M* acts via the adjoint action on $\mathbf{{\mathfrak {m}}}^{\perp _\mathbf{{\mathfrak {k}}}}$. Recall from Sect. 2.1 that $\mathbf{{\mathfrak {m}}}^{\perp _\mathbf{{\mathfrak {k}}}} \cong {{\mathbb {R}}}^n$ and $\text {Ad}(M)|_{\mathbf {{\mathfrak {m}}}^{\perp _\mathbf {{\mathfrak {k}}}}}$ is nothing but the standard action of $\mathrm {SO}(n)$ on ${{\mathbb {R}}}^n$. In the following, we shall write simply $\mathrm {Ad}(M)$ instead of $\mathrm {Ad}(M)|_{\mathbf{{\mathfrak {m}}}^{\perp _\mathbf{{\mathfrak {k}}}}}$. Note that there is a canonical identification

2.35 $$\begin{aligned} K\times _{\mathrm {Ad}(M)}\mathbf {{\mathfrak {m}}}^{\perp _\mathbf {{\mathfrak {k}}}}\cong T(K/M)~ \text { by } ~[k,Y]\mapsto \frac{d}{dt}\Big |_{t=0} ke^{tY}M. \end{aligned}$$

Let $g\in G$ and $\alpha _g: kM \mapsto k_-(gk)M$ be the diffeomorphism on *K*/*M* given by the left-*G*-action, then the derivative $d\alpha _g$ acts on *T*(*K*/*M*). In order to prove our lemma we have to determine how $d\alpha _g$ acts on $K\times _{\mathrm {Ad}(M)}\mathbf{{\mathfrak {m}}}^{\perp _\mathbf{{\mathfrak {k}}}}$ under the identification (2.35). We have for $[k,Y] \in T(K/M)$

2.36 $$\begin{aligned} \begin{aligned} d\alpha _g\left( [k,Y]\right)&= \frac{d}{dt}\Big |_{t=0} k^-(gke^{Yt}) M\\&\cong \left[ k^-(gk),\frac{d}{dt}\Big |_{t=0}k^-(gk)^{-1}k^-(gk\exp (tY))\right] \\&=\left[ k^-(gk),\frac{d}{dt}\Big |_{t=0}k^-\big (a^-(gk)n^-(gk)\exp (tY)n^-(gk)^{-1}a^-(gk)^{-1}\big )\right] \\&=\left[ k^-(gk),\mathrm {pr}_\mathbf{{\mathfrak {k}}}^-\mathrm {Ad}(a^-(gk)n^-(gk))(Y)\right] ,\end{aligned} \end{aligned}$$

where

2.37 $$\begin{aligned} \mathrm {pr}_\mathbf{{\mathfrak {k}}}^-={d} k^-|_e: \mathbf{{\mathfrak {g}}}\rightarrow \mathbf{{\mathfrak {g}}}=\mathbf{{\mathfrak {k}}}\oplus \mathbf{{\mathfrak {a}}}\oplus \mathbf{{\mathfrak {n}}}^- \end{aligned}$$

is the projection onto $\mathbf{{\mathfrak {k}}}$ defined by the opposite Iwasawa decomposition of $\mathbf{{\mathfrak {g}}}$.

We can now proceed by studying for fixed $g\in G$, $k\in K$, $Y\in \mathbf{{\mathfrak {m}}}^{\perp _\mathbf{{\mathfrak {k}}}}$ the element

2.38 $$\begin{aligned} \mathrm {pr}_\mathbf{{\mathfrak {k}}}^-\mathrm {Ad}(a^-(gk)n^-(gk))(Y) \in \mathbf{{\mathfrak {m}}}^{\perp _\mathbf{{\mathfrak {k}}}}. \end{aligned}$$

By the orthogonal Bruhat decomposition $\mathbf{{\mathfrak {g}}}=\mathbf{{\mathfrak {m}}}\oplus \mathbf{{\mathfrak {a}}}\oplus \mathbf{{\mathfrak {n}}}^+\oplus \mathbf{{\mathfrak {n}}}^-$ and the fact that $\mathbf{{\mathfrak {a}}}$ lies in the orthogonal complement of $\mathbf{{\mathfrak {k}}}$ in $\mathbf{{\mathfrak {g}}}$, we have $\mathbf{{\mathfrak {m}}}^{\perp _\mathbf{{\mathfrak {k}}}}\subset \mathbf{{\mathfrak {n}}}^+\oplus \mathbf{{\mathfrak {n}}}^-$, so we can write $Y=Y^++Y^-$ with $Y^\pm \in \mathbf{{\mathfrak {n}}}^\pm$ and $\theta Y^\pm = Y^\mp$. The space $\mathbf{{\mathfrak {n}}}^\pm$ is $\mathrm {Ad}(AN^\pm )$-invariant. Consequently $\mathrm {Ad}(a^-(gk)n^-(gk))(Y^-)\in \mathbf{{\mathfrak {n}}}^-$, so $\mathrm {pr}_\mathbf{{\mathfrak {k}}}^-\mathrm {Ad}(a^-(gk)n^-(gk))(Y^-)=0$ by the opposite Iwasawa decomposition. This shows that only $Y^+$ contributes to (2.38). Let us write $n^-(gk)=\exp (N)$ with $N\in \mathbf{{\mathfrak {n}}}^-$. Then we get

$$\begin{aligned} \mathrm {Ad}(n^-(gk))(Y^+)=e^{\mathrm {ad}(N)}(Y^+)=Y^+ +\underbrace{[N,Y^+]}_{\in \mathbf{{\mathfrak {g}}}_{0}=\mathbf{{\mathfrak {m}}}\oplus \mathbf{{\mathfrak {a}}}}+ \frac{1}{2} \underbrace{[N,[N,Y^+]]}_{\in \mathbf{{\mathfrak {n}}}_-}. \end{aligned}$$

Here we use that $\mathbf{{\mathfrak {g}}}= \mathbf{{\mathfrak {g}}}_0 \oplus \mathbf{{\mathfrak {n}}}^+\oplus \mathbf{{\mathfrak {n}}}^-$ is the root-space decomposition of $\mathbf{{\mathfrak {g}}}=\mathfrak {so}(n+1,1)$ and consequently

$$\begin{aligned} \mathbf{{\mathfrak {n}}}^+\overset{\mathrm {ad}(N)}{\longrightarrow }\mathbf{{\mathfrak {g}}}_0\overset{\mathrm {ad}(N)}{\longrightarrow }\mathbf{{\mathfrak {n}}}^-\overset{\mathrm {ad}(N)}{\longrightarrow }0. \end{aligned}$$

Furthermore, the map $\mathrm {Ad}(a^-(gk))$ acts on $\mathbf{{\mathfrak {n}}}^\pm$ by scalar multiplication with $e^{\pm H^-(gk)}$ and leaves $\mathbf{{\mathfrak {g}}}_0=\mathbf{{\mathfrak {m}}}\oplus \mathbf{{\mathfrak {a}}}$ invariant. The opposite Iwasawa projection $\mathrm {pr}_\mathbf{{\mathfrak {k}}}^-$ maps $\mathbf{{\mathfrak {n}}}^-$ to 0 and the space $\mathbf{{\mathfrak {g}}}_{0}$ onto $\mathbf{{\mathfrak {m}}}$. However, the Lie algebra element considered in (2.38) is by construction in $\mathbf{{\mathfrak {m}}}^{\perp _\mathbf{{\mathfrak {k}}}}$. We therefore arrive at

$$\begin{aligned} {\mathrm {pr}}_\mathbf {{\mathfrak {k}}}^-\mathrm {Ad}(a^-(gk)n^-(gk))(Y)=\mathrm {pr}_\mathbf {{\mathfrak {k}}}^-\big ( e^{ H^-(gk)}Y^+\big ) . \end{aligned}$$

Writing

$$\begin{aligned} Y^+ =\underbrace{Y^+ + \theta Y^+}_{\in \mathbf {{\mathfrak {k}}}}- \underbrace{\theta Y^+}_{\in \mathbf {{\mathfrak {n}}}^-} ~~ \text { reveals } ~~ \mathrm {pr}_\mathbf {{\mathfrak {k}}}^-\mathrm {Ad}(a^-(gk)n^-(gk))(Y) =e^{H^-(gk)}Y. \end{aligned}$$

In summary, we have proved that

2.39 $$\begin{aligned} d\alpha _g ([k,Y])=\big [k^-(gk), e^{H^-(gk)}Y\big ]. \end{aligned}$$

Finally, note that $T(K/M)\cong K\times _{\mathrm {Ad}(M)}\mathbf{{\mathfrak {m}}}^{\perp _\mathbf{{\mathfrak {k}}}}$ induces for each $p\in \{1,2,\ldots \}$ an isomorphism $\Lambda ^p T^*(K/M)\cong K\times _{\Lambda ^p\mathrm {Ad}^*(M)}\Lambda ^p(\mathbf{{\mathfrak {m}}}^{\perp _\mathbf{{\mathfrak {k}}}})^*$. Under that isomorphism, a *p*-form $s\in \Gamma ^\infty (\Lambda ^p T^*(K/M))$ corresponds to a section ${\hat{s}}\in \Gamma ^\infty (K\times _{\Lambda ^p\mathrm {Ad}^*(M)}\Lambda ^p(\mathbf{{\mathfrak {m}}}^{\perp _\mathbf{{\mathfrak {k}}}})^*)$, and by our above computations the pullback action $gs\equiv (g^{-1})^*s$ of an element $g\in G$ on *s* corresponds to the following action on ${\hat{s}}$:

2.40 $$\begin{aligned} \begin{aligned} \overline{(g {\hat{s}})}(k)(X_1,\ldots ,X_p)&=\overline{{\hat{s}}}(k^-(g^{-1}k))(e^{H^-(g^{-1}k)}X_1,\ldots ,e^{H^-(g^{-1}k)}X_p)\\&=e^{pH^-(g^{-1}k)}\overline{{\hat{s}}}(k^-(g^{-1}k))(X_1,\ldots ,X_p)\qquad \forall \; X_1,\ldots ,X_p\in \mathbf{{\mathfrak {n}}}^\pm ,\;k\in K.\end{aligned} \end{aligned}$$

Recalling the definition (2.21) of the principal series representations, and taking into account that the pullback action of *G* on *p*-currents as well as the principal series representations of *G* on distributional sections of $\Lambda ^pT^*(K/M)$ are the continuous extensions of the respective actions on smooth *p*-forms, the proof is complete. $\square$

For the definition of his Poisson transform Gaillard generalizes his setting to currents with values in complex line bundles $D^s\rightarrow K/M$ parametrized by a complex number $s\in {{\mathbb {C}}}$. Let us recall their construction [Gai86, Sect. 2.2]: It is based on a *G*-invariant function^12^

2.41 $$\begin{aligned} Q:G/K\times K/M\times G/K\rightarrow {{\mathbb {C}}}\setminus \{0\},\qquad Q(gK,kM,eK)=\Vert D(V^{-1}_{gK}\circ V_{eK})|_{kM} \Vert ,\nonumber \\ \end{aligned}$$

where Gaillard’s “application visuelle” $V_{gK}: S^*_{gK}(G/K)\rightarrow K/M$, $gK\in G/K$, is defined by

$$\begin{aligned} V_{gK}: \{{\tilde{g}}M:{\tilde{g}}K=gK\}=S^*_{gK}(G/K)\rightarrow K/M,\qquad {\tilde{g}}M \mapsto k^-({\tilde{g}}) M. \end{aligned}$$

A straightforward calculation similar to the proof of Lemma 2.8 shows that

2.42 $$\begin{aligned} Q(gK,kM,eK)=e^{H^-(g^{-1}k)}, \end{aligned}$$

which gives us by the *G*-invariance of *Q* for a general element $({\tilde{g}}K,kM,gK)\in G/K\times K/M\times G/K$:

2.43 $$\begin{aligned} Q({\tilde{g}}K,kM,gK)&=Q(g(g^{-1}{\tilde{g}}K,k^-(g^{-1}k)M,eK))\nonumber \\&=Q(g^{-1}{\tilde{g}}K,k^-(g^{-1}k)M,eK)\nonumber \\&=e^{H^-({\tilde{g}}^{-1}g k^-(g^{-1}k))}. \end{aligned}$$

With these preparations, let us now turn to Gaillard’s definition of the line bundle $D^s$ over *K*/*M*: Introduce an equivalence relation $\sim _s$ on $G/K\times K/M\times {{\mathbb {C}}}$ by

$$\begin{aligned} (gK,kM,z)\sim _s ({\tilde{g}}K,{\tilde{k}}M,{\tilde{z}}) \iff kM= & {} \tilde{k}M,\;\\ {\tilde{z}}= & {} Q({\tilde{g}}K,kM,gK)^{-s} z=e^{-sH^-({\tilde{g}}^{-1}g k^-(g^{-1}k))}z, \end{aligned}$$

and declare $D^s:=G/K\times K/M\times {{\mathbb {C}}}/\sim _s$ with bundle projection $[gK,kM,z]\mapsto kM$. The bundle is a homogeneous *G*-bundle by defining the *G* action as

$$\begin{aligned} g'[gK,kM,z]:=[g'gK,g'(kM),z]=[g'gK,k^-(g'k)M,z]. \end{aligned}$$

The stabilizer subgroup of $eM\in K/M$ with respect to the left-*G*-action on *K*/*M* is $MAN^-$ and the action of the stabilizer group on the fiber of $D^s$ over *eM* is

$$\begin{aligned}{}[manK , eM, z] = [eK, eM, e^{-sH(mank^-(n^{-1}a^{-1}m^{-1}))}z]= [eK, eM, e^{-s\log (a)}z]. \end{aligned}$$

If we define the $MAN^-$-representation $\sigma _s$ by $man\mapsto e^{-s\log (a)} \in {{\mathbb {C}}}$ then we can identify $D^s$ with the associated line bundle $G \times _{\sigma _s}{{\mathbb {C}}}\rightarrow G/(MAN^-)\cong K/M$. Thus the *G*-action on sections of this homogenous bundle is equivalent to the principal series representation $\pi ^{s}_{\mathbb 1}$, where $\mathbb 1$ denotes the trivial *M*-representation on ${{\mathbb {C}}}$. By Lemma 2.8 we know that the pullback action on *p*-currents is equivalent to $\pi ^{p-n/2}_{\tau _p}$, so the action of *G* on $D^s$-valued currents is equivalent to $\pi ^{p-n/2}_{\tau _p}\otimes \pi ^{s}_{\mathbb {1}}$ which is equivalent to $\pi ^{p+s-n/2}_{\tau _p}$.

## Non-Constant Curvature Perturbations

We now address the question how the equality $m_{{\mathcal {L}}_X, X^\perp }(0)=b_1({\mathcal {M}})$ for constant negative curvature manifolds behaves under perturbations of the Riemannian metric and also under more general perturbations of the vector field *X* that do not (only) result from metric perturbations. Throughout this section, let ${\mathcal {M}}$ be a closed orientable manifold admitting a hyperbolic metric and $\Gamma ^\infty (\mathrm {S}^2(T^*{{\mathcal {M}}}))$ the space of symmetric two-tensors endowed with the Fréchet topology. Let $\mathscr {R}_{{\mathcal {M}},<0}\subset \Gamma ^\infty (\mathrm {S}^2(T^*{{\mathcal {M}}}))$ be the open subset of Riemannian metrics of negative sectional curvature. For any Riemannian metric  on ${{\mathcal {M}}}$, we write  for the geodesic vector field on the unit sphere bundle  with respect to . In order to study perturbations of the vector field , we consider $S$ as a Riemannian manifold equipped with the metric  induced by the Sasaki metric on $T^*{\mathcal {M}}$ with respect to  and define the $\mathrm {C}^1$-norm on  by3.1where  is the Levi-Civita connection with respect to  and we denoted the metric obtained by extending  to the tensor bundle  also by .

With this notation at hand, we can prepare the proof of our main Theorem 0.3 which will be given on page 17. As already indicated in the introduction, we essentially reduce the proof to two steps: Lemma 3.1 provides a local upper bound for the multiplicity of an arbitrary resonance, while Lemma 3.2 provides global lower bounds for the resonance zero. Finally, Lemma 3.4 relates the multiplicities of the resonance zero on general and on perpendicular one-forms.

### Lemma 3.1

For each $\lambda \in {{\mathbb {C}}}$ and each  there is an open set $U\subset \mathscr {R}_{{\mathcal {M}},<0}$ containing  and a constant $\delta >0$ such that for all  and all  with  one has

### Proof

Fix some reference metric  for the rest of the proof and let  be some arbitrary Riemannian metric on ${{\mathcal {M}}}$. Then the diffeomorphism , , fulfills3.2For a vector field , consider its pushforward . By the naturality of the Lie derivative with respect to pullbacks, the following diagram commutes: 
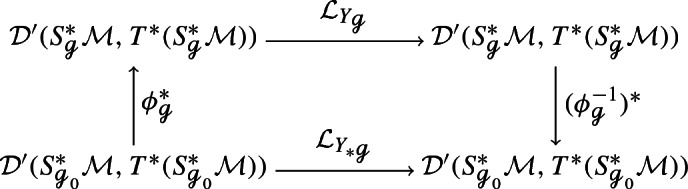


By comparing the pushforward connection  with  using the Koszul formula, one checks thatwith a constant  that depends continuously on  with respect to the Fréchet topology on $\Gamma ^\infty (\mathrm {S}^2(T^*{{\mathcal {M}}}))$. Furthermore, the geodesic vector fields  and  fulfillThus, for every $\varepsilon >0$ we can find an open set $U\subset {\mathscr {R}}_{{\mathcal {M}},<0}$ containing  and a $\delta >0$ such that3.3Choosing $\varepsilon$ small enough, the structural stability (see [KM73, Thm. A]) of the Anosov property of vector fields on the -independent manifold  allows us to assume from now on that  is Anosov for all . Then also  is Anosov for all . Indeed, the Anosov splitting of  is obtained by applying  to the Anosov splitting of . In view of the commutative diagram above one has3.4Given some $\lambda \in {{\mathbb {C}}}$ we now apply the perturbation result [Bon18], which says that on every closed manifold the resonances of all Anosov vector fields *Y* that are $\mathrm {C}^1$-close to a given Anosov vector field $Y_0$ can be defined as eigenvalues in certain Hilbert spaces that depend only on $Y_0$ and not on *Y*, so that the change of the multiplicity of $\lambda$ in this fixed Hilbert space can be measured as *Y* varies near $Y_0$. The results of [Bon18] generalize easily to a vector-valued situation (for vector bundles that do not vary with the vector field *Y*) by replacing the scalar quantization map in [Bon18, Eq. (2)] by a vector-valued quantization map. The correspondingly generalized [Bon18, Cor. 2] then implies that there is a $c >0$ such that all  with  fulfill3.5Choosing $\varepsilon <c$ in (3.3), we can put  in (3.5) for each , and by (3.4) the proof is finished. $\square$

A second ingredient to Theorem 0.3 is a very general lower bound on the multiplicity of the resonance zero:

### Lemma 3.2

  For some Riemannian metric  on ${{\mathcal {M}}}$, let  be an Anosov vector field. Thenand if there is a one-form  on  with  and , then

### Remark 3.3

Lemma 3.2 remains true, with the same proof, if  is replaced by an arbitrary closed oriented manifold ${\mathcal {N}}$ and  by an Anosov vector field $Y\in \Gamma ^\infty (T{\mathcal {N}})$.

### Proof of Lemma 3.2

Fix some Riemannian metric  on ${{\mathcal {M}}}$. Dang-Rivière [DR19c] proved that for every Anosov vector field forms a finite-dimensional complex whose cohomology is isomorphic to the de Rham cohomology of . This implies , proving the first inequality. Now suppose that there is a one-form  with  and . By the wave front characterization of resonant states (1.3) we then know that  for each locally constant function *c* on  (thus each element in the 0-th de Rham cohomology). Since  if $c\ne 0$, the second inequality follows. $\square$

We can now prove Theorem 0.3:

### Proof of Theorem 0.3

Assume that $\dim {{\mathcal {M}}}\ne 3$ and let  be a metric of constant negative curvature. Then we can apply Proposition 2.1 and (3.7) with , , and  the canonical contact form on  to get3.6Now, if  is any Riemannian metric on ${{\mathcal {M}}}$, then by [CS50, (4.1)] one has , and we also have  because $\dim {{\mathcal {M}}}\ge 2$ (otherwise ${{\mathcal {M}}}$ would not admit metrics of negative sectional curvature). Thus, it suffices to apply the local upper bound from Lemma 3.1 for $\lambda =0$ and the global lower bounds from Lemma 3.2 to finish the proof of Theorem 0.3. $\square$

Finally, in order to get a statement involving resonance multiplicities on the bundle , one can use the following basic result:

### Lemma 3.4

For some Riemannian metric  on ${{\mathcal {M}}}$, let  be an Anosov vector field. If there is a one-form  on  with , , and , then3.7

### Proof

As  and , we can uniquely decompose every  into  where  and thus $u_\perp$ is a distributional section of . We have for some $J\in {{\mathbb {N}}}$3.8since ,  implies . Using Cartan’s magic formula one checks , so the wave front characterization of resonant states (1.3) implies  and . The latter space is of dimension $b_0({\mathcal {M}})$ as it consists of the locally constant functions (cf. the end of the proof of Lemma 2.7), so we get (3.7). $\square$

## Footnotes

1 The methods of Fried to study the Ruelle zeta function generalize to locally symmetric spaces culminating in the recent work of Shen [She18].

2 We would like to point out that an analogous statement also holds for Morse-Smale flows [DR19b, DR19a, DR18, DR19c] and in these cases the spectral complex defined by the Pollicott-Ruelle resonances is actually isomorphic to the Morse complex. Consequently, the spectral complex of Dang and Rivière can be considered as a generalization of the Morse complex to Anosov flows.

3 See the paragraph below (1.3) for the definition of “having no Jordan block” and the footnote in Prop. 2.1 for the other terms used here.

4 It has been noted in [DGRS19, Remark 5] (without detailing the proof) that the statement of Proposition 0.1 can alternatively be obtained by a zeta factorization argument similar to [DGRS19, Proposition 7.7] based on the work of Bunke and Olbrich [BO95].

5 See (3.1) for the definition of the $\mathrm {C}^1$-norm used here. For small  the vector field  is Anosov by the structural stability of the Anosov property [KM73], so that the Pollicott-Ruelle resonances of  are well-defined.

6 I.e., a Riemannian manifold of constant sectional curvature $-1$. Fixing the curvature at $-1$ is a common convention. By trivial rescaling arguments all results in this paper involving the resonance 0 remain true if the metric is multiplied by a positive constant.

7 I.e., there exists $\delta >0$ such that ${\mathcal {L}}_X$ acting on $X^\perp$ has no resonances with real part in the interval $(-\delta ,\infty )$ except the resonance zero.

8 Here the subscript 0 indicates the identity component.

9 We use a simplified (non-standard) notation and follow Olbrich’s convention as in [Olb95, between Satz 2.8 and Satz 2.9]. In [Kna86, p. 169], the definition differs from ours in such a way that $\lambda$ is replaced by $-\lambda$. Furthermore recall that we identified $\mathbf{{\mathfrak {a}}}\cong {{\mathbb {R}}}$ in Sect. 2.1.

10 Since the geodesic flow preserves the Liouville measure on $S^*{{\mathcal {M}}}$ and the norm on the bundle $E_-$, one can show that $\nabla _X$ is antisymmetric in $\mathrm{L}^2$ and one can write down an explicit formula for the $\mathrm{L}^2$-resolvent $(\nabla _X+\lambda )^{-1}$ when $\mathrm {Re}\,\lambda >0$, see e.g. [DG16, (1.10)].

11 We thank Colin Guillarmou for suggesting the slick argument.

12 Here *G* acts on all three factors in the domain $G/K\times K/M\times G/K$ by left multiplication.
